# Predictive value of complete blood cell count-based inflammatory markers for peritonitis risk in peritoneal dialysis patients: A multicenter cohort study

**DOI:** 10.1371/journal.pone.0354120

**Published:** 2026-07-22

**Authors:** Xue Li, Wenlong Qiu, Qingdong Xu, Yueqiang Wen, Xianfeng Wu, Xiaojiang Zhan, Fenfen Peng, Xiaoyang Wang, Juan Wu, Ning Su, Xiaoran Feng, Xingming Tang, Qian Zhou, Na Tian

**Affiliations:** 1 Department of Nephrology, General Hospital of Ningxia Medical University, Yinchuan, China; 2 Ningxia Clinical Research Center of Kidney Disease, Yinchuan, China; 3 Department of Nephrology, Jiangmen Central Hospital, Jiangmen, China; 4 Department of Nephrology, The Second Affiliated Hospital of Guangzhou Medical University, Guangzhou, China; 5 Department of Nephrology, Affiliated Sixth People’s Hospital, Shanghai Jiao Tong University, Shanghai, China; 6 Department of Nephrology, The First Affiliated Hospital of Nanchang University, Nanchang, China; 7 Department of Nephrology, Zhujiang Hospital, Southern Medical University, Guangzhou, China; 8 Department of Nephrology, The First Affiliated Hospital of Zhengzhou University, Zhengzhou, China; 9 Department of Nephrology, Urology & Nephrology Center, Zhejiang Provincial People’s Hospital (Affiliated People’s Hospital), Hangzhou Medical College, Hangzhou, Zhejiang, China; 10 Department of Hematology, The Sixth Affiliated Hospital of Sun Yat-Sen University, Guangzhou, China; 11 Department of Nephrology, Jiujiang No. 1 People’s Hospital, Jiujiang, China; 12 Department of Nephrology, DongGuan SongShan Lake Tungwah Hospital, Dongguan, China; 13 Department of Medical Statistics, Clinical Trials Unit, The First Affiliated Hospital, Sun Yat-sen University, Guangzhou, China; University of Diyala College of Medicine, IRAQ

## Abstract

**Background:**

Peritonitis is a serious complication of peritoneal dialysis (PD). Inflammatory indices derived from routine complete blood count (CBC) parameters—including the pan-immune inflammatory value (PIV), systemic immune-inflammatory index (SII), platelet-to-lymphocyte ratio (PLR), neutrophil-to-lymphocyte ratio (NLR), monocyte-to-lymphocyte ratio (MLR), and platelet-to-monocyte ratio (PMR)—have shown prognostic value in various diseases. However, their comparative utility in predicting PD-associated peritonitis (PDAP) remains unclear. This multicenter cohort study aimed to evaluate and compare these indices to identify the best predictor of PDAP.

**Methods:**

We retrospectively enrolled 2,036 PD patients from 10 centers. The associations between inflammatory markers (PIV, SII, PLR, NLR, MLR, PMR) and peritonitis risk were analyzed using restricted cubic splines. Optimal cut-offs were determined by ROC analysis. Survival differences were assessed using Kaplan–Meier curves and log-rank tests. Independent predictors were identified via multivariate Cox regression, with model discrimination evaluated by the C-index. Subgroup analyses were conducted by gender, age, body mass index (BMI), diabetes, albumin, PD vintage, and residual renal function.

**Results:**

The median age was 51.0 years, 55.01% were male, and median dialysis vintage was 49.47 months. Diabetes prevalence was 21.02%. Over the follow-up, 147 patients (7.22%) developed peritonitis. Among the indices evaluated, PIV, SII, and PLR showed significant nonlinear associations with peritonitis risk (all *P* < 0.05). Adjusted hazard ratios were 2.004 for PIV, 2.144 for SII, and 2.063 for PLR. Adjusted C-indices were 0.67 (PIV), 0.70 (SII), and 0.70 (PLR). No significant interactions were found in subgroup analyses.

**Conclusion:**

Elevated PIV, SII, and PLR levels at PD initiation independently predict higher peritonitis risk. Although their discriminative ability is moderate, these routine, cost-effective indices may aid risk stratification and help identify patients needing closer monitoring or preventive interventions.

## Introduction

Among the options for end-stage renal disease (ESRD), peritoneal dialysis (PD) offers several advantages, including operational simplicity, lower cost, and better preservation of residual renal function, making it a preferred choice for many patients [1].However, PD-associated peritonitis(PDAP)is a frequent and serious complication that can lead to catheter removal, technique failure, and even death [2]. The annual incidence of peritonitis is approximately 0.26 episodes per patient-year. Approximately 30% of patients experience at least one peritonitis episode within the first year of PD [3]. Although the incidence rates of PDAP have declined in recent years, it is a major cause of morbidity, mortality, and treatment discontinuation [4]. Therefore, early identification of high-risk patients and effective intervention can aid in improving patient outcomes and reducing disease burden.

In this context, inflammatory markers derived from peripheral blood cell counts have attracted growing interest for their prognostic utility. Among these, composite indices that integrate multiple cell lineages—such as the pan-immune-inflammatory value (PIV), which combines neutrophil, platelet, monocyte, and lymphocyte counts, and the systemic immune-inflammation index (SII), based on neutrophil, lymphocyte, and platelet counts—offer a more comprehensive reflection of systemic immune-inflammatory status than single-ratio markers. Theoretically, these multicellular indices capture complex interactions between innate and adaptive immunity, as well as thrombotic pathways, thereby providing a broader and potentially more accurate risk assessment [5–7]. In contrast, simpler ratios, including the platelet-to-lymphocyte ratio (PLR), monocyte-to-lymphocyte ratio (MLR), neutrophil-to-lymphocyte ratio (NLR), and platelet-to-monocyte ratio (PMR), while readily available and clinically useful in various settings [8–13], may lack the integrated information afforded by composite indices. Notably, PLR and SII have already been applied in the setting of PDAP [9], and MLR and NLR have shown associations with mortality and cardiovascular events in PD patients [10,11]. Theoretically, PIV offers superior discriminative capacity because it simultaneously reflects the pro-inflammatory burden (neutrophils, platelets, monocytes) and the adaptive immune reserve (lymphocytes), thereby providing a more comprehensive assessment of systemic inflammation and immune dysregulation.

Therefore, the extent to which these CBC-derived inflammatory indices can predict PDAP remains largely unknown. To address this gap, we conducted a multicenter retrospective study to systematically assess and compare the predictive performance of several such indices, with the goal of identifying the most effective biomarker for clinical risk stratification in PDAP.

## Subjects and methods

### Subjects

This multicenter, observational cohort study enrolled 6,366 patients from 10 PD centers in China. The inclusion criteria were as follows:(patients aged ≥18 years) patients who had undergone PD for at least three months. The exclusion criteria were as follows: (1) acute inflammatory diseases (e.g., pneumonia, enteritis) within the first three months PD initiation; (2) a history of malignancy, except for non-metastatic thyroid cancer or breast cancer in complete remission; (3) active autoimmune diseases; (4) current use of medications that may interfere with CBC results, such as aspirin, corticosteroids, or immunosuppressants; and (5) missing essential laboratory data, including neutrophil, platelet, lymphocyte, and monocyte counts, as well as hemoglobin, high-sensitivity C-reactive protein, and serum albumin levels. The final cohort comprised 2,036 patients. PDAP was diagnosed according to the International Society for Peritoneal Dialysis (ISPD) guidelines. A diagnosis was established when at least two of the following three criteria were met: (1) clinical signs and symptoms of peritonitis, such as abdominal pain and/or turbid dialysate; (2) after a dwell time of at least 2 hours, a dialysis effluent white blood cell count of > 100 cells/μL (or > 0.1 × 10⁹ cells/L), with > 50% polymorphonuclear leukocytes; (3) positive microbial culture of the dialysate [14].

### Methods

The multicenter, retrospective cohort study was performed according to the principles of the Declaration of Helsinki. Ethical approval and waiver of informed consent were obtained from the institutional review boards (IRBs) or ethics committees of all participating centers: the Medical Ethics Committee of the First Affiliated Hospital of Nanchang University (Approval No. IIT[2025]临伦审第852号), the Ethics Committee of the Sixth Affiliated Hospital of Sun Yat-Sen University (Approval Nos. 2021ZSLYEC-177 and E2021082), the Medical Research Ethics Committee of General Hospital of Ningxia Medical University (Approval No. KYLL-2022–0496), and the Clinical Research and Application Ethics Committee of the Second Affiliated Hospital of Guangzhou Medical University (Acceptance No. 2024-hg-ks-11). Because the study involved analysis of anonymized historical clinical data and posed no more than minimal risk to participants, all committees waived the requirement for individual written informed consent. Clinical data were retrospectively collected from the medical records of patients who initiated PD between July 21, 1998, and May 31, 2023. The retrospective data extraction for this study was performed between July 1, 2025, and October 1, 2025. Patient data meeting the inclusion criteria were retrieved from the databases during this period. The extracted dataset contained no direct personal identifiers (e.g., name, national ID, hospital admission number, or contact information); each patient was assigned a unique anonymized code. Throughout the analysis and manuscript preparation, the research team had no access to identifiable participant information. This study adheres to the Strengthening the Reporting of Observational Studies in Epidemiology (STROBE) guidelines for cohort studies.

### Baseline data collection

The baseline data, including demographic characteristics, comorbidities, medication use, and laboratory parameters derived from electronic medical records, were collected at PD initiation. All assessments were completed within the first three months of therapy, which served as a stable baseline period for obtaining laboratory parameters (including CBC and derived inflammatory markers). Patients who developed peritonitis during this three-month baseline period were excluded. For participants with multiple laboratory tests, the initial result was designated as the baseline value.

The following demographic variables were retrieved: age, gender, body mass index (BMI), smoking history, alcohol consumption, and medical history of diabetes (International Classification of Diseases, 10th Revision [ICD-10] codes E10–E14), hypertension (ICD-10 codes I10–I15), hyperlipidemia (ICD-10 code E78), and cardiovascular disease (CVD). CVD was defined as a diagnosis of coronary artery disease (ICD-10 codes I20–I25), heart failure (ICD-10 code I50), cerebrovascular disease (ICD-10 codes I60–I69), or peripheral arterial disease (ICD-10 codes I70–I79) documented by a physician.

The following laboratory parameters were examined: white blood cell count, red blood cell count, neutrophil count, lymphocyte count, platelet count, monocyte count, hemoglobin level, fasting blood glucose, high-sensitivity C-reactive protein, aspartate aminotransferase, alanine aminotransferase, serum albumin, serum creatinine, blood urea nitrogen, uric acid, triglycerides, total cholesterol, serum phosphate, serum calcium, and serum potassium. All laboratory measurements were performed using standard assays at the respective PD center laboratories.

Inflammatory markers were derived from routine blood tests and calculated as follows:

-
$\text{PIV}=[\text{neutrophil count }({\text{10}}^{\text{9}}/\text{L})\times \text{platelet count }({\text{10}}^{\text{9}}/\text{L})\times \text{monocyte count }({\text{10}}^{\text{9}}/\text{L})]/\text{lymphocyte count }({\text{10}}^{\text{9}}/\text{L})$

-
$\text{SII}=[\text{neutrophil count }({\text{10}}^{\text{9}}/\text{L})\times \text{platelet count }({\text{10}}^{\text{9}}/\text{L})]/\text{lymphocyte count }({\text{10}}^{\text{9}}/\text{L})$

-
$\text{PLR}=\text{platelet count }({\text{10}}^{\text{9}}/\text{L})/\text{lymphocyte count }({\text{10}}^{\text{9}}/\text{L})$

-
$\text{MLR}=\text{monocyte count }({\text{10}}^{\text{9}}/\text{L})/\text{lymphocyte count }({\text{10}}^{\text{9}}/\text{L})$

-
$\text{NLR}=\text{neutrophil count }({\text{10}}^{\text{9}}/\text{L})/\text{lymphocyte count }({\text{10}}^{\text{9}}/\text{L})$

-
$\text{PMR}=\text{platelet count }({\text{10}}^{\text{9}}/\text{L})/\text{monocyte count }({\text{10}}^{\text{9}}/\text{L})$

Patient medical history was obtained from the medical record homepage, while supplementary data were collected from hospitalization records and physician order forms. Attending physicians at each PD center reviewed the electronic medical records. Trained research staff entered the data into the database. Data accuracy was verified by trained graduate students. Patients were scheduled for follow-up assessments at their respective centers once every 1–3 months. Trained nurses conducted monthly telephone follow-ups to monitor patients’ overall health status.

### Follow-up

The follow-up period was from July 21, 1998, to May 31, 2023. Patients were monitored until the occurrence of a study endpoint event, which was defined as death, kidney transplantation, initiation of hemodialysis, transfer to another center, or loss to follow-up.

### Statistical analysis

All statistical analyses were performed using SPSS (version 27.0), R software (version 4.5.1), and Zstats software (version 1.0; www.zstats.net). Differences were considered significant at *P* < 0.05(two-sided test). Continuous variables are presented as mean ± standard deviation (SD) or median (interquartile range, IQR), and categorical variables as n (%). Normality was assessed with the Kolmogorov–Smirnov test. Missing skewed data were median-imputed.

Nonlinear associations between inflammatory markers and peritonitis risk were identified using restricted cubic splines. ROC-derived optimal cut-offs stratified patients into high- and low-level groups. Kaplan–Meier curves and log-rank tests compared time-to-event outcomes.

Cox proportional hazards models evaluated peritonitis risk factors. Model 1: unadjusted; Model 2: adjusted for CVD, diabetes, sex, age, and albumin; Model 3: adjusted for Model 2 variables and smoking, hyperlipidemia, hs-CRP, hemoglobin, and total Kt/V. The results are reported as HRs (95% CIs). Model discrimination was assessed using Harrell’s C-index.

Subgroup and interaction analyses were performed based one gender, age, BMI, diabetes, albumin, PD vintage, and residual renal function.

## Results

**Fig 1:**
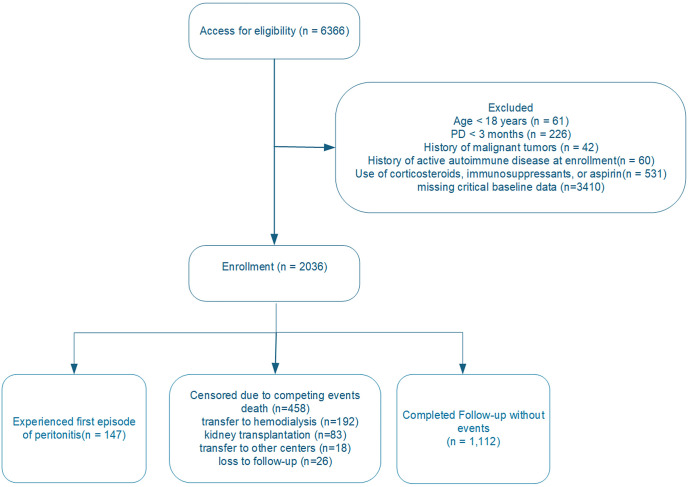
Flowchart of participant enrollment and exclusion criteria.

This study enrolled 2,036 patients. The median follow-up duration was 49.47 months (IQR: 22.23–97.50). During the follow-up period, the first peritonitis episode occurred in 147 patients. The data of 777 patients were censored due to the following reasons: death (n = 458), transfer to hemodialysis (n = 192), kidney transplantation (n = 83), transfer to other centers (n = 18), and loss to follow-up (n = 26) (Fig 1).

### Baseline patient characteristics

Of the 2036 patients, 147 (7.2%) developed peritonitis. Compared to the non-peritonitis group, these patients had significantly higher levels of PLR, SII, MLR, NLR, and PIV (all p < 0.05), and lower levels of albumin, peritoneal dialysis vintage, residual renal function, serum creatinine, AST, ALT, triglycerides, and potassium (all p < 0.05). Clinically, the peritonitis group had a higher prevalence of smoking, alcohol use, and hypertension, but a lower prevalence of cardiovascular disease (all p < 0.05).

Among the six inflammatory markers screened, only PMR showed no significant difference (p = 0.987). The remaining five markers (SII, PIV, NLR, PLR, MLR) were therefore included in subsequent model comparisons (Table 1).

**Table 1:** Demographic and laboratory data of the study population.

Variables	Total (n = 2036)	Without peritonitis (n = 1889)	With peritonitis (n = 147)	Statistic	P
Age, M (Q_1_, Q_3_)	51.00 (40.75, 62.00)	51.00 (40.00, 62.00)	54.00 (46.00, 63.00)	Z = −1.82	0.069
Gender, Male, n (%)	1120 (55.01)	1041 (55.11)	79 (53.74)	χ² = 0.10	0.748
Smoking History, n (%)	93 (4.57)	78 (4.13)	15 (10.20)	χ² = 11.55	<.001
Drinking History, n (%)	52 (2.55)	43 (2.28)	9 (6.12)	χ² = 6.63	0.010
Diabetes, n (%)	428 (21.02)	391 (20.70)	37 (25.17)	χ² = 1.64	0.200
Hypertension, n (%)	1527 (75.00)	1400 (74.11)	127 (86.39)	χ² = 10.97	<.001
CVD History, n (%)	322 (15.82)	308 (16.30)	14 (9.52)	χ² = 4.71	0.030
Hyperlipidemia, n (%)	31 (1.52)	28 (1.48)	3 (2.04)	χ² = 0.03	0.855
PD Vintage, M (Q_1_, Q_3_), Month	49.47 (22.23, 97.50)	54.37 (23.43, 101.03)	23.20 (9.47, 38.67)	Z = −0.35	0.724
BMI, M (Q_1_, Q_3_)	21.88 (19.82, 24.22)	21.88 (19.81, 24.22)	21.88 (20.12, 24.23)	Z = −0.22	0.987
Total Kt/V, M (Q_1_, Q_3_)	2.13 (1.85, 2.56)	2.13 (1.85, 2.56)	2.06 (1.66, 2.51)	Z = −0.81	0.417
RRF, M (Q_1_, Q_3_), ml/min	3.53 (1.91, 5.53)	3.65 (2.03, 5.67)	2.26 (0.61, 3.94)	Z = −5.30	<.001
WBC, M (Q_1_, Q_3_)	6.36 (5.07, 7.88)	6.34 (5.06, 7.83)	6.38 (5.17, 8.89)	Z = −1.82	0.068
PLT, M (Q_1_, Q_3_)	202.00 (155.00, 256.25)	201.00 (153.00, 255.00)	214.00 (172.00, 270.50)	Z = −2.49	0.013
Hemoglobin, M (Q_1_, Q_3_), g/L	96.00 (78.00, 113.00)	96.00 (78.00, 113.00)	98.00 (82.00, 111.00)	Z = −0.98	0.329
Albumin, M (Q_1_, Q_3_), g/L	35.90 (31.87, 39.50)	36.00 (32.00, 39.60)	34.10 (29.25, 38.25)	Z = −3.48	<.001
hs-CRP, M (Q_1_, Q_3_), mg/L	3.30 (1.26, 10.98)	3.30 (1.26, 10.79)	3.48 (1.22, 14.58)	Z = −0.53	0.594
ALP, M (Q₁, Q_3_), U/L	74.73 (59.00, 93.00)	75.00 (59.00, 93.00)	72.00 (57.50, 96.00)	Z = −0.66	0.509
Scr, M (Q_1_, Q_3_), μmol/L	794.40 (609.75, 937.40)	787.00 (600.00, 942.80)	794.40 (754.00, 848.50)	Z = −2.29	0.022
BUN, M (Q_1_, Q_3_), mmol/L	19.57 (15.40, 22.90)	19.57 (15.30, 23.20)	19.57 (17.05, 19.75)	Z = −0.53	0.595
UC, M (Q_1_, Q_3_), mol/L	396.00 (330.75, 476.00)	396.00 (330.00, 476.00)	397.00 (333.00, 484.00)	Z = −0.54	0.590
FBG, M (Q_1_, Q_3_), mmol/L	4.92 (4.30, 5.54)	4.90 (4.30, 5.54)	5.54 (4.50, 5.54)	Z = −1.51	0.132
AST, M (Q_1_, Q_3_), U/L	18.15 (14.00, 24.83)	18.63 (14.00, 25.00)	17.00 (13.00, 22.00)	Z = −2.28	0.022
ALT, M (Q_1_, Q_3_), U/L	14.00 (10.00, 22.00)	14.00 (10.00, 22.00)	12.00 (8.20, 20.00)	Z = −2.23	0.026
TC, M (Q_1_, Q_3_), mmol/L	4.55 (3.72, 5.46)	4.57 (3.72, 5.47)	4.50 (3.71, 5.37)	Z = −0.42	0.675
Triglyceride, M (Q_1_, Q_3_), mmol/L	1.40 (0.99, 1.98)	1.40 (1.00, 1.99)	1.28 (0.85, 1.84)	Z = −2.32	0.020
Calcium, M (Q_1_, Q_3_), mmol/L	2.15 (1.98, 2.31)	2.15 (1.98, 2.31)	2.16 (1.98, 2.33)	Z = −1.06	0.288
Potassium, M (Q_1_, Q_3_), mmol/L	4.08 (3.60, 4.62)	4.10 (3.60, 4.67)	3.78 (3.40, 4.38)	Z = −4.19	<.001
Phosphorus, M (Q_1_, Q_3_), mmol/L	1.64 (1.30, 1.97)	1.62 (1.30, 1.97)	1.72 (1.41, 2.02)	Z = −1.80	0.073
IPTH, M (Q_1_, Q_3_), pg/ml	239.50 (121.44, 394.95)	235.90 (122.04, 388.00)	310.40 (105.65, 452.90)	Z = −1.45	0.148
PIV, M (Q_1_, Q_3_)	278.97 (153.42, 486.69)	273.55 (151.73, 479.03)	390.08 (211.36, 615.74)	Z = −3.82	<.001
PLR, M (Q_1_, Q_3_)	156.56 (113.21, 216.90)	155.82 (112.87, 214.63)	170.66 (122.41, 261.97)	Z = −2.44	0.015
SII, M (Q_1_, Q_3_)	673.01(427.95,1044.90)	662.76 (425.93, 1028.10)	860.00 (490.58, 1288.01)	Z = −3.41	<.001
NLR, M (Q_1_, Q_3_)	3.39 (2.44, 4.75)	3.38 (2.42, 4.70)	3.78 (2.54, 5.59)	Z = −2.02	0.044
MLR, M (Q_1_, Q_3_)	0.33 (0.24, 0.45)	0.33 (0.24, 0.44)	0.36 (0.28, 0.52)	Z = −3.07	0.002
PMR, M (Q_1_, Q_3_)	476.34 (340.88, 670.66)	475.00 (340.91, 670.02)	501.82 (341.92, 679.88)	Z = −0.02	0.987

M: Median, Q_1_: 1st Quartile, Q_3_: 3rd Quartile. BMI: body mass index; CVD: cardiovascular disease; RRF: residual renal function; WBC: white blood cell count; PLT: platelet count;RBC: red blood cell count; NEUT: neutrophil count; MONO:monocyte count; LYMPH: lymphocyte count; hs-CRP: high-sensitivity c-reactive protein; ALP: alkaline phosphatase; Scr:serum creatinine; BUN:blood urea nitrogen; UC: uric acid; AST:aspartate aminotransferase; ALT: alanine aminotransferase; TC:total cholesterol; FBG: fasting blood glucose; iPTH: intact parathyroid hormone

### Analysis of the correlation of inflammatory markers with peritonitis risk, and their predictive value

#### Assessment of nonlinear associations.

**Fig 2:**
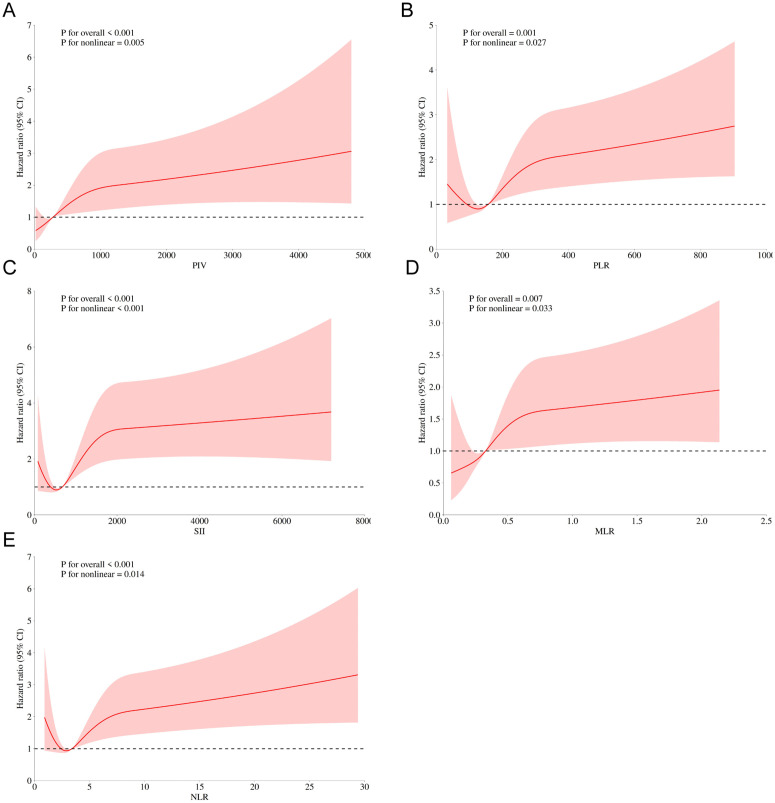
Hazard ratios (HRs) and 95% confidence intervals for peritonitis risk in peritoneal dialysis (PD) patients were assessed using restricted cubic spline models for platelet-to-lymphocyte ratio (PLR), systemic immune-inflammation index (SII), pan-immune-inflammatory value (PIV), monocyte-to-lymphocyte ratio (MLR), and neutrophil-to-lymphocyte ratio (NLR).

RCS analysis (Fig 2, A–E) revealed significant nonlinear associations between peritonitis risk and five inflammatory markers—PLR, SII, PIV, MLR, and NLR (all P < 0.05 for overall and nonlinear terms). The hazard ratio reached 1 at the following reference values: PLR = 156.56, SII = 673.01, PIV = 278.97, MLR = 0.33, and NLR = 3.39.

#### Predictive performance based on nonlinear associations.

ROC analysis revealed that all inflammatory markers showed limited discriminatory power for peritonitis (area under the curve [AUC], 0.55–0.59). PIV demonstrated the highest predictive value (AUC = 0.59, p < 0.001). The markers displayed distinct sensitivity–specificity profiles: MLR had high sensitivity (0.73) but low specificity (0.39), whereas PLR showed high specificity (0.86) but low sensitivity (0.27). SII presented a more balanced profile (sensitivity 0.46, specificity 0.72). NLR did not reach statistical significance (AUC = 0.55, p = 0.062). Due to its very low specificity, MLR was considered clinically impractical owing to a high false-positive rate. Consequently, both NLR and MLR were excluded from further analyses (Table 2).

**Table 2:** Receiver operating characteristic (ROC) curve analysis of inflammatory markers for predicting peritonitis in peritoneal dialysis (PD) patients.

Variables	AUC (95%CI)	Sensitivity (95%CI)	Specificity (95%CI)	Cut off	P Value
PLR	0.56(0.51-0.61)	0.27 (0.19 - 0.34)	0.86 (0.84 - 0.87)	260.09	0.023
SII	0.58 (0.53-0.64)	0.46 (0.38 - 0.54)	0.72 (0.70 - 0.74)	963.41	0.001
PIV	0.59(0.55-0.64)	0.52(0.44-0.60)	0.66(0.64-0.68)	378.72	<0.001
MLR	0.58 (0.53-0.62)	0.73 (0.66 - 0.80)	0.39(0.37 - 0.41)	0.29	0.02
NLR	0.55(0.50-0.60)	0.55(0.47-0.63)	0.56(0.54-0.59)	3.65	0.062

To translate the identified nonlinear relationships into clinically applicable tools, optimal cut-off values for the remaining markers were determined using ROC curve analysis. Patients were subsequently stratified into high- level and low-level groups based on the cut-off values of PLR, SII, and PIV.

**Fig 3:**
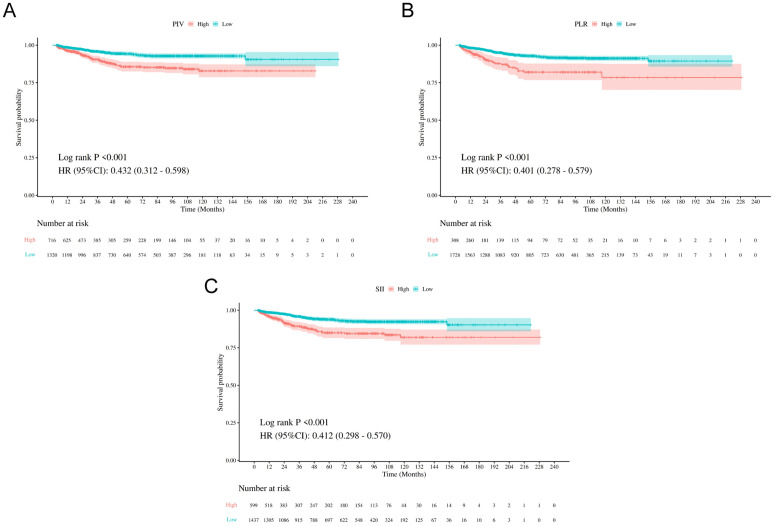
Kaplan-Meier curves illustrating the cumulative incidence of peritonitis in peritoneal dialysis (PD) patients stratified according to the pan-immune-inflammatory value (PIV), platelet-to-lymphocyte ratio (PLR), and systemic immune-inflammation index (SII) levels. The curves were compared using the log-rank test (p < 0.001).

Kaplan-Meier analysis revealed that elevated levels of PIV, PLR, and SII significantly increased the risk of peritonitis in PD patients (log-rank test, p < 0.001; Fig 3A-3C).

As shown in Table 3, multivariate Cox regression analyses demonstrated that elevated levels of PIV, PLR, and SII were significantly associated with an increased risk of peritonitis in model 1, model 2, and model 3 (all p < 0.005). The stability of the HRs after sequential adjustment for clinical and laboratory confounders confirmed that these indices are independent risk factors for peritonitis.

**Table 3:** Association of pan-immune-inflammatory value (PIV), platelet-to-lymphocyte ratio (PLR), and systemic immune-inflammation index (SII) with peritonitis risk in peritoneal dialysis (PD) patients: Hazard ratios (HRs), 95% confidence intervals (CIs), and P-values in different adjustment models.

Group	Model 1 HR (95%CI)	P-value	Model 2 HR (95%CI)	P-value	Model 3 HR (95%CI)	P-value
**PIV**						
Low-level	Reference					
High-level	2.314 (1.674-3.200)	<0.001	2.087 (1.502-2.899)	<0.001	2.004 (1.439-2.789)	<0.001
**PLR**						
Low-level	Reference					
High-level	2.493 (1.728-3.599)	<0.001	2.136 (1.471-3.101)	<0.001	2.063 (1.415-3.006)	<0.001
**SII**						
Low-level	Reference					
High-level	2.426 (1.753-3.357)	<0.001	2.181 (1.569-3.033)	<0.001	2.144 (1.536-2.992)	<0.001

**Model 1:** Unadjusted.

**Model 2:** Adjusted for cardiovascular disease, diabetes, sex, age, and albumin.

**Model 3:** Adjusted for Model 2 variables plus smoking, hyperlipidemia, high-sensitivity C-reactive protein, hemoglobin, and total Kt/V.

#### C-indices of PIV, PLR, and SII.

**Fig 4:**
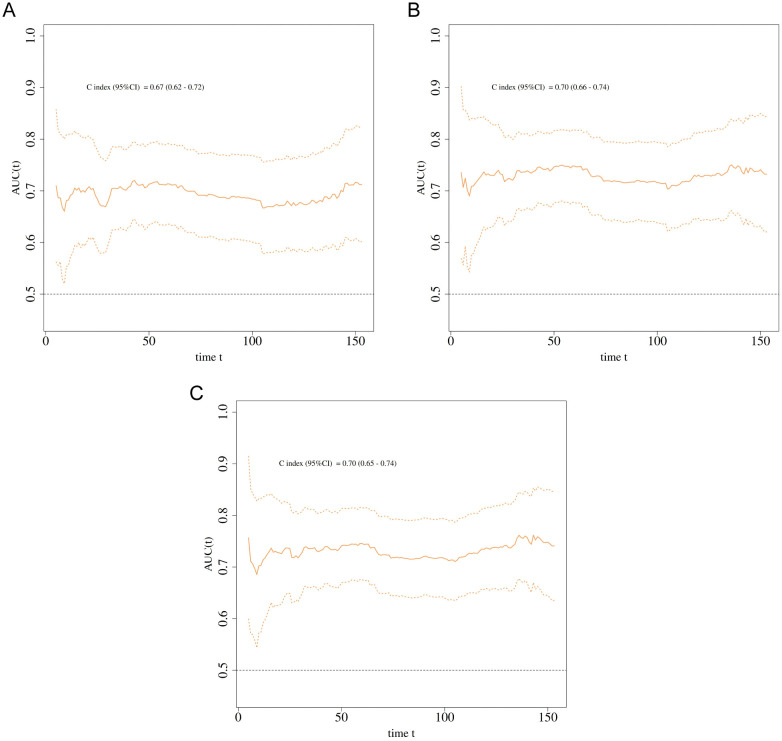
Predictive performance of PIV, PLR and SII for peritonitis in PD patients as measured by the C-index.

Following multivariable adjustment, all inflammatory markers demonstrated similar predictive ability for peritonitis, with C-indices of 0.67 (95% CI: 0.62–0.72) for PIV, 0.70 (0.66–0.74) for PLR, and 0.70 (0.65–0.74) for SII. Although their performance was comparable, the highest C-index (0.70) still exceeded the AUC of any single marker in the prior univariable ROC analysis, suggesting that these indices may retain potential utility for clinical risk stratification (Fig 4A–4C).

#### Subgroup analysis.

**Fig 5:**
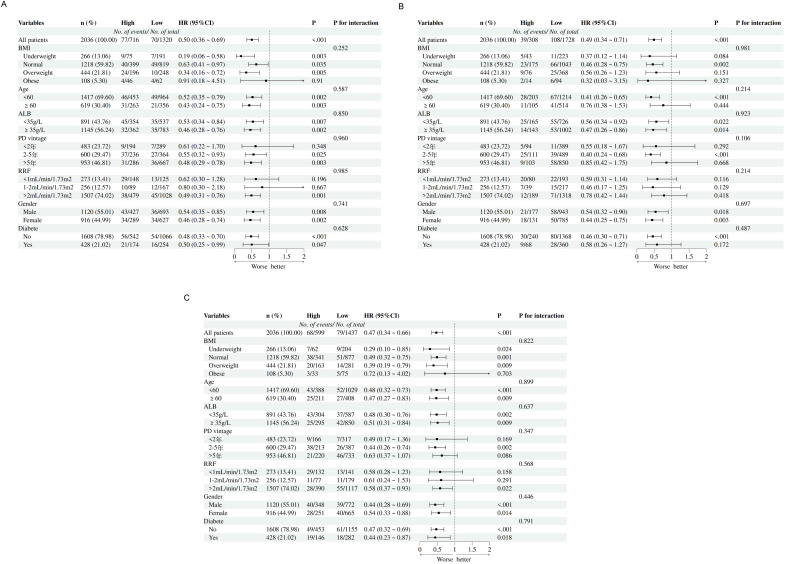
Subgroup analysis of the associations of pan-immune-inflammatory value (PIV) (A), platelet-to-lymphocyte ratio (PLR) (B), and systemic immune-inflammation index (SII) (C) with peritonitis risk in peritoneal dialysis (PD) patients.

Subgroup analyses were performed to assess the consistency of the correlation of high PIV, PLR, and SII levels with the risk of peritonitis based on gender, age (< 60 or ≥ 60 years), BMI (underweight, healthy, or overweight), diabetes, serum albumin level (< 35 or ≥ 35 g/L), PD vintage (< 2, 2–5, or > 5 years), and RRF (< 1, 1–2, or > 2 mL/min/1.73m^2^). The forest plots (Fig 5A–5C) did not reveal significant interaction effects. This indicates that these associations were consistent in all examined subgroups.

## Discussion

This multicenter retrospective cohort study demonstrated that elevated levels of PIV, SII, and PLR at PD initiation are independently associated with an increased risk of subsequent peritonitis. Comparative analysis revealed that PIV did not exhibit superior predictive performance when compared with the PLR or SII indices. These findings suggest that CBC-derived inflammatory indices serve as useful adjuncts for risk stratification in patients undergoing PD.

Routine CBC test, an accessible and economical test, provides valuable insights into the systemic inflammatory status of patients [15,16]. Neutrophils, which belong to the primary leukocyte subgroup in acute inflammatory responses, exhibit clinically significant quantitative changes. Peripheral neutrophil counts, which markedly increase during inflammation, are widely used for diagnosing and monitoring inflammatory conditions [15,17]. In addition to facilitating coagulation by providing a phospholipid surface, activated platelets release various inflammatory mediators that recruit and activate monocytes and neutrophils [18], This can induce vascular endothelial injury and increase vascular permeability, amplifying inflammatory responses and edema [19]. Lymphocytes, which have critical roles in adaptive immunity, have protective and immunomodulatory functions [20]. Under chronic inflammatory conditions, lymphocyte counts often decrease due to immune microenvironment disruption. Thus, lymphocyte counts are an objective indicator of persistent inflammation and immune competence [21].

The SII integrates neutrophil, platelet, and lymphocyte counts. SII elevation is typically driven by increased neutrophil and platelet counts coupled with decreased lymphocytes, reflecting systemic inflammatory activation and immune imbalance. The PIV is a relatively novel biomarker that incorporates neutrophil, monocyte, platelet, and lymphocyte counts. Thus, the PIV enables a comprehensive assessment of inflammatory severity and immune dysregulation when compared with SII and PLR. Previous studies have reported that PIV serves as a robust prognostic predictor in patients with advanced cancer receiving cytotoxic chemotherapy, immunotherapy, or targeted therapy [22]. Limited studies have reported the clinical value of PIV in non-oncological contexts. One study revealed that the pre-treatment PIV levels can serve as a valuable biomarker for predicting long-term survival outcomes in patients with colorectal cancer and have important clinical implications for personalized treatment strategies [23]. Pre-treatment PIV levels are also reported to predict pathological complete response and survival outcomes in patients with breast cancer, outperforming NLR, MLR, and PLR in predicting pathological complete response [24].

In contrast to PIV, SII, and PLR, the platelet-to-monocyte ratio (PMR) showed no significant association with peritonitis risk (p = 0.987). Several explanations may account for this finding. First, monocytes play a dual role in peritoneal defense. A growing body of evidence indicates that while monocyte-derived inflammatory macrophages promote peritoneal fibrosis and disease progression, tissue-resident macrophages are rather considered anti-fibrotic [25]. Long-term PD exposure alters resident peritoneal macrophage phenotype, leading to loss of homeostatic and anti-inflammatory functionality while enhancing inflammatory responses to external stimuli, rendering patients sensitive to peritonitis and subsequent fibrosis [25]. Thus, the net effect of monocytes may depend on their interaction with other immune cells rather than on their absolute count alone. PIV explicitly incorporates neutrophil count (neutrophil × platelet × monocyte/ lymphocyte), whereas PMR treats monocytes as an isolated denominator and therefore fails to capture this complex modulation. Second, neutrophils and monocytes exhibit intricate functional interactions in peritoneal inflammation. During septic peritonitis, neutrophils suppress peritoneal inflammatory monocytes through IL-10-mediated regulation, with in vitro experiments confirming that monocyte suppression is mediated by neutrophil-derived IL-10 [26]. This suggests that the inflammatory potential of monocytes is actively modulated by the concomitant neutrophil response—a biological nuance that PIV captures through its multiplicative structure, but PMR, as a simple ratio, does not.

Third, PMR’s lack of predictive value may also reflect methodological limitations of the ratio itself. A low PMR could reflect either low platelet count or high monocyte count; the latter might indicate a protective inflammatory response rather than a pathogenic one, leading to opposing clinical interpretations from the same numerical value. Moreover, the optimal balance between platelets and monocytes may vary across individuals, and a simple ratio may not capture the complex interplay among multiple cell lineages. Consistent with this notion, a recent study in PD patients demonstrated that SII —a composite inflammatory index structurally analogous to PIV—was an independent risk factor for frequent PD-associated peritonitis, and combining SII with C-reactive protein (CRP)/high-density lipoprotein cholesterol (HDL-C) yielded better predictive performance than single markers [27]. This finding supports our conclusion that the predictive signal carried by monocytes is only apparent when modulated by neutrophil and lymphocyte counts, as operationalized in the PIV formula.

The clinical utility of a predictive marker depends critically on its sensitivity–specificity balance. In our cohort, PLR’s low sensitivity (27%) implies that using this cut-off alone would fail to identify most patients who subsequently develop peritonitis, potentially leading to under-recognition of high-risk individuals. Conversely, its high specificity (86%) means that a positive result is unlikely to be a false alarm. For SII and PIV, the moderate sensitivity and specificity suggest they may serve as adjunctive rather than standalone tests. Importantly, we explicitly excluded MLR from further analyses because its very low specificity (39%) would result in an unacceptably high false-positive rate, causing unnecessary patient anxiety and potentially unwarranted clinical interventions. Therefore, in current clinical practice, we recommend that these markers be interpreted in combination with other clinical parameters, and that dynamic changes over time be considered to improve predictive accuracy.

Cheuk-Chun Szeto et al. [28] summarized preventive strategies against PDAP, with a focus on PD equipment, patient training, and nursing practices. The authors outlined specific primary and secondary prevention measures. Advances in therapeutic protocols and clinical guidelines have not markedly mitigated the incidence of peritonitis, warranting continued research and clinical practice improvement [28]. The current management of peritonitis majorly involves antibiotic therapy and surgical intervention. The clinical challenges associated with this management strategy are the increasing prevalence of antibiotic resistance and frequent delays in diagnosis [29]. These limitations often compromise treatment efficacy, underscoring the urgent need for developing reliable predictive tools. These predictive tools are critical for enabling early identification and timely intervention in high-risk patients [30].

To facilitate clinical translation, the correlation of these novel markers to established, traditional risk factors for peritonitis, such as hypoalbuminemia, hypokalemia, lower RRF, diabetes, and older age, must be determined [31,32]. In the study cohort, the peritonitis group exhibited significantly lower albumin, RRF, and potassium levels. PIV, SII, and PLR were independent predictors after adjustment for several key confounders. This suggests that PIV, SII, and PLR capture a distinct aspect of risk, potentially related to subclinical systemic inflammation. However, the standalone predictive accuracy of PIV, SII, and PLR was modest. The C-indices for PIV (0.67), SII (0.70), and PLR (0.70) showed modest discrimination. Despite this, all three indices retained independent predictive value in the fully adjusted model (Model 3), indicating that their prognostic information is not driven by confounding. Nevertheless, a C-index of 0.67–0.70 is suboptimal for a stand-alone risk stratification tool. Hence, these peripheral blood inflammatory indices should be considered risk modifiers that complement conventional models rather than independent risk stratifiers. Therefore, the clinical utility of PIV, SII, and PLR lies in their ability to refine risk assessment when combined with conventional clinical evaluation. For example, a patient with borderline hypoalbuminemia and a markedly elevated SII might be prioritized for intensified training and monitoring.

This study has several strengths, including its large, multicenter design and long-term follow-up. However, this study is associated with several limitations. First, the retrospective design can lead to selection bias and does not account for all confounding factors. This study adjusted for available clinical and laboratory variables. However, the effects of residual confounding factors, such as detailed connection systems, exit-site care practices, and patient adherence cannot be excluded. Second, the high rate of competing events (e.g., death, transfer to hemodialysis) is an important methodological consideration. This study used standard Cox regression. Future studies must use competing risk models to provide further validation of the risk estimates. Third, the single optimal cut-off approach is clinically pragmatic but simplifies the continuous, nonlinear relationships. Fourth, microbiological culture results were not systematically available in this retrospective cohort, which limits our ability to assess whether the predictive performance of CBC-derived markers differs by pathogen type. Future prospective studies should incorporate standardized microbial sampling to address this question. Fifth, our primary analysis excluded patients who developed peritonitis within the first three months after PD initiation. This exclusion was based on a well-established clinical rationale—the early ‘break-in’ period is characterized by unique, transient inflammatory drivers that differ from steady-state peritonitis risk [33,34]. Nevertheless, we acknowledge that this approach may introduce immortal time bias and selection bias, and our findings are most applicable to peritonitis risk assessment after the initial three-month stabilization period. Future prospective studies with serial biomarker collection starting from PD catheter implantation are needed to validate our findings in the very early post-PD period. Sixth, we did not collect serial complete blood count data during follow-up and therefore could not perform time-dependent Cox regression analyses. A single baseline measurement, while reflecting the patient’s initial inflammatory set-point at PD transition, may not capture subsequent changes in inflammatory status over a median follow-up of 49 months. We acknowledge that inflammatory markers can fluctuate over time in response to intercurrent infections, changes in dialysis prescription, or nutritional status. Nevertheless, evaluating the predictive value of baseline markers is a clinically meaningful and methodologically accepted approach in prognostic research, and our findings demonstrate that even a one-time measurement provides useful risk stratification. The lack of repeated measurements precludes assessment of whether dynamic changes in these markers (e.g., a rising PIV or SII) add further predictive value. Future prospective studies with standardized serial sampling (e.g., every 3–6 months) are needed to validate the utility of time-dependent inflammatory marker monitoring and to determine whether incorporating longitudinal data improves peritonitis risk prediction over baseline assessment alone. Seventh, we acknowledge that net reclassification improvement (NRI) was not evaluated in the present study. Although Model 3 demonstrated independent predictive value for PIV, SII, and PLR, whether adding these indices to conventional risk factors significantly improves net reclassification remains unknown. This omission is a clear limitation. In subsequent external validation or prospective studies, we plan to perform NRI analyses to determine whether models incorporating PIV, SII, or PLR together with established clinical factors yield clinically meaningful net reclassification gains. Finally, the use of median imputation for a small proportion of missing data, while preserving sample size, may not fully address potential bias from non-random missing data [35]. Future prospective, multi-center studies involving more diverse populations and rigorous external validation are warranted to confirm the reproducibility and generalizability of the findings of this study.

In conclusion, PIV, SII, and PLR measured at PD initiation are independently associated with long-term peritonitis risk. Comparative analysis of the performance of PIV, SII, and PLR revealed that the simple indices SII and PLR may be adequate for clinical consideration. These markers have potential applications as integrative components of a broad risk assessment strategy. Future prospective research should focus on developing and validating a composite clinical score that combines these inflammatory indices with traditional risk factors to optimize early identification and personalized preventive care for patients on PD.

## References

[1] Li PK-T, Chow KM, Van de Luijtgaarden MWM, Johnson DW, Jager KJ, Mehrotra R, et al. Changes in the worldwide epidemiology of peritoneal dialysis. Nat Rev Nephrol. 2017;13(2):90–103. doi: 10.1038/nrneph.2016.181 28029154

[2] Dzekova-Vidimliski P, Nikolov IG, Gjorgjievski N, Selim G, Trajceska L, Stojanoska A, et al. Peritoneal Dialysis-Related Peritonitis: Rate, Clinical Outcomes and Patient Survival. Pril (Makedon Akad Nauk Umet Odd Med Nauki). 2021;42(3):47–55. doi: 10.2478/prilozi-2021-0034 35032377

[3] Flythe JE, Watnick S. Dialysis for Chronic Kidney Failure: A Review. Jama. 2024;332(18):1559–73. doi: 10.1001/jama.2024.16338 39356511

[4] Nardelli L, Scalamogna A, Castellano G. Utility of ultrasonographic examination in catheter-related infections in peritoneal dialysis: a clinical approach. J Nephrol. 2023;36(7):1751–61. doi: 10.1007/s40620-023-01589-w 36939999 PMC10543158

[5] Lin F, Zhang L-P, Xie S-Y, Huang H-Y, Chen X-Y, Jiang T-C, et al. Pan-Immune-Inflammation Value: A New Prognostic Index in Operative Breast Cancer. Front Oncol. 2022;12:830138. doi: 10.3389/fonc.2022.830138 35494034 PMC9043599

[6] Akcam TI, Tekneci AK, Turhan K, Duman S, Cuhatutar S, Ozkan B, et al. Prognostic value of systemic inflammation markers in early stage non-small cell lung cancer. Sci Rep. 2025;15(1):33886. doi: 10.1038/s41598-025-08683-y 41028061 PMC12484583

[7] Hu B, Yang X-R, Xu Y, Sun Y-F, Sun C, Guo W, et al. Systemic immune-inflammation index predicts prognosis of patients after curative resection for hepatocellular carcinoma. Clin Cancer Res. 2014;20(23):6212–22. doi: 10.1158/1078-0432.CCR-14-0442 25271081

[8] Yang S, Liang G, Sun J, Yang L, Fu Z, Sun W, et al. Higher baseline platelet and preoperative platelets to lymphocytes ratio was associated with a higher incidence of axillary node pathologic complete response after neoadjuvant chemotherapy in HER2-low breast cancer: a retrospective cohort study. Front Oncol. 2025;15:1437677. doi: 10.3389/fonc.2025.1437677 40161379 PMC11949971

[9] Su N, Zheng Y, Zhang X, Tang X, Tang L-W, Wang Q, et al. Platelet-to-lymphocyte ratio and the first occurrence of peritonitis in peritoneal dialysis patients. BMC Nephrol. 2022;23(1):415. doi: 10.1186/s12882-022-03038-5 36585653 PMC9803258

[10] Yang Y, Xu Y, Lu P, Zhou H, Yang M, Xiang L. The prognostic value of monocyte-to-lymphocyte ratio in peritoneal dialysis patients. Eur J Med Res. 2023;28(1):152. doi: 10.1186/s40001-023-01073-y 37038225 PMC10084613

[11] Lu X, Wang S, Zhang G, Xiong R, Li H. High Neutrophil-to-Lymphocyte Ratio is a Significant Predictor of Cardiovascular and All-Cause Mortality in Patients Undergoing Peritoneal Dialysis. Kidney Blood Press Res. 2018;43(2):490–9. doi: 10.1159/000488696 29627842

[12] Batur AF, Aydogan MF, Kilic O, Korez MK, Gul M, Kaynar M, et al. Comparison of De Ritis Ratio and other systemic inflammatory parameters for the prediction of prognosis of patients with transitional cell bladder cancer. Int J Clin Pract. 2021;75(4):e13743. doi: 10.1111/ijcp.13743 32991771

[13] Wu J, Fu L, Deng Z, Li H, Zhong L, Gao R, et al. A study of changes in hematologic parameters in patients with migraine. Clin Exp Immunol. 2025;219(1):uxae113. doi: 10.1093/cei/uxae113 39660838 PMC11773810

[14] Li PK, Szeto CC, Piraino B, de Arteaga J, Fan S, Figueiredo AE, et al. ISPD Peritonitis Recommendations: 2016 Update on Prevention and Treatment. Perit Dial Int. 2016;36(5):481–508. doi: 10.3747/pdi.2016.00078 27282851 PMC5033625

[15] Guthrie GJK, Charles KA, Roxburgh CSD, Horgan PG, McMillan DC, Clarke SJ. The systemic inflammation-based neutrophil-lymphocyte ratio: experience in patients with cancer. Crit Rev Oncol Hematol. 2013;88(1):218–30. doi: 10.1016/j.critrevonc.2013.03.010 23602134

[16] Kamrani F, Mohammadzadeh M, Sobhani SR, Khorasanchi Z. Phase angle and water cell distribution as inflammation indicators linked to hematological markers across BMI categories. Sci Rep. 2025;15(1):16147. doi: 10.1038/s41598-025-98430-0 40341652 PMC12062412

[17] Turner N, Wong H-L, Templeton A, Tripathy S, Whiti Rogers T, Croxford M, et al. Analysis of local chronic inflammatory cell infiltrate combined with systemic inflammation improves prognostication in stage II colon cancer independent of standard clinicopathologic criteria. Int J Cancer. 2016;138(3):671–8. doi: 10.1002/ijc.29805 26270488

[18] Sloan AR, Lee-Poturalski C, Hoffman HC, Harris PL, Elder TE, Richardson B, et al. Glioma stem cells activate platelets by plasma-independent thrombin production to promote glioblastoma tumorigenesis. Neurooncol Adv. 2022;4(1):vdac172. doi: 10.1093/noajnl/vdac172 36452274 PMC9700385

[19] Zubair F, McMahon J, Kryklyas G, Wicks C. Systemic inflammatory response in predicting outcomes of patients undergoing curative resection for oral squamous cell carcinoma. Br J Oral Maxillofac Surg. 2022;60(5):589–95. doi: 10.1016/j.bjoms.2021.10.017 35248409

[20] Braun A-S, Vomstein K, Reiser E, Tollinger S, Kyvelidou C, Feil K, et al. NK and T Cell Subtypes in the Endometrium of Patients with Recurrent Pregnancy Loss and Recurrent Implantation Failure: Implications for Pregnancy Success. J Clin Med. 2023;12(17):5585. doi: 10.3390/jcm12175585 37685653 PMC10488644

[21] Man M-A, Davidescu L, Motoc N-S, Rajnoveanu R-M, Bondor C-I, Pop C-M, et al. Diagnostic Value of the Neutrophil-to-Lymphocyte Ratio (NLR) and Platelet-to-Lymphocyte Ratio (PLR) in Various Respiratory Diseases: A Retrospective Analysis. Diagnostics (Basel). 2021;12(1):81. doi: 10.3390/diagnostics12010081 35054248 PMC8774859

[22] Şahin AB, Cubukcu E, Ocak B, Deligonul A, Oyucu Orhan S, Tolunay S, et al. Low pan-immune-inflammation-value predicts better chemotherapy response and survival in breast cancer patients treated with neoadjuvant chemotherapy. Sci Rep. 2021;11(1):14662. doi: 10.1038/s41598-021-94184-7 34282214 PMC8289916

[23] Li J, Pang H, Sun H, Liu X. Prognostic significance of the pretreatment pan-immune-inflammation value in colorectal cancer patients: an updated meta-analysis. Front Oncol. 2025;15:1599075. doi: 10.3389/fonc.2025.1599075 40777110 PMC12328146

[24] Su Z, Tang J, He Y, Zeng WH, Yu Q, Cao XL, et al. Pan‑immune‑inflammation value as a novel prognostic biomarker in nasopharyngeal carcinoma. Oncol Lett. 2024;27(6):252. doi: 10.3892/ol.2024.14385 38646495 PMC11027095

[25] Helmke A, Nordlohne J, Balzer MS, Dong L, Rong S, Hiss M, et al. CX3CL1-CX3CR1 interaction mediates macrophage-mesothelial cross talk and promotes peritoneal fibrosis. Kidney Int. 2019;95(6):1405–17. doi: 10.1016/j.kint.2018.12.030 30948201

[26] Ocuin LM, Bamboat ZM, Balachandran VP, Cavnar MJ, Obaid H, Plitas G, et al. Neutrophil IL-10 suppresses peritoneal inflammatory monocytes during polymicrobial sepsis. J Leukoc Biol. 2011;89(3):423–32. doi: 10.1189/jlb.0810479 21106642 PMC3040467

[27] Tang J, Wang D, Chen Y, Feng J. The association between new inflammation markers and frequent peritoneal dialysis-associated peritonitis. BMC Nephrol. 2024;25(1):81. doi: 10.1186/s12882-024-03496-z 38443857 PMC10916203

[28] Szeto CC, Li PK. Peritoneal Dialysis-Associated Peritonitis. Clin J Am Soc Nephrol. 2019;14(7):1100–5. doi: 10.2215/cjn.14631218 31068338 PMC6625612

[29] Li PK-T, Chow KM, Cho Y, Fan S, Figueiredo AE, Harris T, et al. ISPD peritonitis guideline recommendations: 2022 update on prevention and treatment. Perit Dial Int. 2022;42(2):110–53. doi: 10.1177/08968608221080586 35264029

[30] Brown EA, Blake PG, Boudville N, Davies S, de Arteaga J, Dong J, et al. International Society for Peritoneal Dialysis practice recommendations: Prescribing high-quality goal-directed peritoneal dialysis. Perit Dial Int. 2020;40(3):244–53. doi: 10.1177/0896860819895364 32063219

[31] Szeto C-C, Ng JK-C, Fung WW-S, Chan GC-K, Cheng PM-S, Law M-C, et al. Excessive risk and poor outcome of hospital-acquired peritoneal dialysis-related peritonitis. Clin Kidney J. 2022;15(11):2107–15. doi: 10.1093/ckj/sfac164 36325003 PMC9613437

[32] Muthucumarana K, Howson P, Crawford D, Burrows S, Swaminathan R, Irish A. The Relationship Between Presentation and the Time of Initial Administration of Antibiotics With Outcomes of Peritonitis in Peritoneal Dialysis Patients: The PROMPT Study. Kidney International Reports. 2016;1(2):65–72. doi: 10.1016/j.ekir.2016.05.003 29142915 PMC5678844

[33] Poulain L, Bechade C, Lanot A, Ficheux M, Guillouet S, Lobbedez T, et al. Early-Onset Peritonitis and Outcomes of Peritoneal Dialysis: A Cohort Study with Data from the RDPLF. Am J Nephrol. 2025;56(4):391–402. doi: 10.1159/000542835 39900007

[34] Wu H, Huang R, Yi C, Wu J, Guo Q, Zhou Q, et al. Risk Factors for Early-Onset Peritonitis in Southern Chinese Peritoneal Dialysis Patients. Perit Dial Int. 2016;36(6):640–6. doi: 10.3747/pdi.2015.00203 27147289 PMC5174871

[35] Zhang Z. Missing data imputation: focusing on single imputation. Ann Transl Med. 2016;4(1):9. doi: 10.3978/j.issn.2305-5839.2015.12.38 26855945 PMC4716933

