# Supplementary material for: Predictive value of complete blood cell count-based inflammatory markers for peritonitis risk in peritoneal dialysis patients: A multicenter cohort study
Source: PLoS One. 2026 Jul 22;21(7):e0354120. doi: 10.1371/journal.pone.0354120 (PMC13390817; doi:10.1371/journal.pone.0354120)
Supplement: Attachment — Submitted filename: Response to Reviewers.docx [file pone.0354120.s001.docx]

**Response to Reviewers**
Manuscript ID: PONE-D-26-05780
Title: Predictive Values of Complete Blood Cell Count-Based Inflammatory Markers for Peritonitis Risk in Peritoneal Dialysis Patients: A Multicenter Cohort Study
Corresponding Author: Na Tian, MD, PhD

Dear Dr. Serdoncillo and the Editorial Team,

Thank you for your feedback. We have revised the manuscript as requested.

**Editor’s comment:**
Please clarify whether the study began at the same time at each study site. If not, report the dates when data were accessed for research purposes for each site in DD/MM/YYYY format.

**Our response:**
We confirm that all centers initiated retrospective patient data collection on the same date.

In the revised manuscript, we have added the following sentence to **lines 150–152** of the Methods section:

“All centers initiated retrospective patient data collection on the same date (01/07/2025) and completed data extraction by 01/10/2025.”

This date represents the exact period when data were accessed for research purposes at all participating centers. All centers began concurrently; therefore, no site-specific breakdown is required.

Sincerely,
Na Tian, MD, PhD
Corresponding Author
