# Supplementary material for: Predictive value of complete blood cell count-based inflammatory markers for peritonitis risk in peritoneal dialysis patients: A multicenter cohort study
Source: PLoS One. 2026 Jul 22;21(7):e0354120. doi: 10.1371/journal.pone.0354120 (PMC13390817; doi:10.1371/journal.pone.0354120)
Supplement: Attachment — Submitted filename: Response_to_Reviewers_auresp_1.docx [file pone.0354120.s002.docx]

Dear Academic Editor and Reviewers,

We sincerely thank you for your thoughtful and constructive comments on our manuscript. We have carefully addressed all concerns point by point and revised the manuscript accordingly. Below we provide our detailed responses. All modifications have been highlighted in the revised manuscript with track changes.

**Responses to Academic Editor**

***Editor’s comment 1:*** *Recheck the sentence "white blood cell count >100/μL" page 8 Line 133.*

**Response:**

We thank the Editor for this careful observation. We have rechecked the sentence on page 8, line 133. The original description “white blood cell count >100/μL” was a typographical error. We have revised the sentence to: “**after a dwell time of at least 2 hours, a dialysis effluent white blood cell count of > 100 cells/μL (or > 0.1 × 10⁹ cells/L), with > 50% polymorphonuclear leukocytes**” [1-5]. Due to other revisions in the manuscript, this sentence is now located on **Page 7, Lines 127–129** in the tracked-changes manuscript.

[1] Hsu CK, Lee CC, Chen YT, Ting MK, Sun CY, Chen CY, Hsu HJ, Chen YC, Wu IW. Multidisciplinary predialysis education reduces incidence of peritonitis and subsequent death in peritoneal dialysis patients: 5-year cohort study. PLoS One. 2018 Aug 23;13(8):e0202781. doi: 10.1371/journal.pone.0202781. PMID: 30138478; PMCID: PMC6107258.

[2] Yamaguchi M, Obayashi T, Kobayashi N, Izumi N, Nagai M, Nobata H, Asai A, Kamiya K, Sugiyama H, Kinashi H, Banno S, Ando M, Imaizumi T, Kubo Y, Katsuno T, Ishimoto T, Ito Y. Association between reduced left ventricular ejection fraction and peritoneal dialysis related peritonitis: a single center retrospective cohort study in Japan. Sci Rep. 2023 Dec 20;13(1):22697. doi: 10.1038/s41598-023-49744-4. PMID: 38123633; PMCID: PMC10733284.

[3] Liu Y, Zhao W, Lu H, Liu A. Risk factors and microbiological characteristics of bacterial peritonitis in patients undergoing peritoneal dialysis. BMC Nephrol. 2026 Jan 7;27(1):88. doi: 10.1186/s12882-025-04711-1. PMID: 41501722; PMCID: PMC12870372.

[4] Nie S, Zhang Q, Chen R, Lin L, Li Z, Sun Y, Huang J, Feng Z, Cao X, Ye K, Tang L, Zhou J, Cai G, Chen X. Rapid detection of pathogens of peritoneal dialysis-related peritonitis, especially in patients who have taken antibiotics, using metagenomic next-generation sequencing: a pilot study. Ren Fail. 2023;45(2):2284229. doi: 10.1080/0886022X.2023.2284229. Epub 2023 Nov 29. PMID: 38031397; PMCID: PMC11001327.

[5] Yang Q, Ren X, Fang X, Ding J. The efficacy of cuff-shaving combined with negative pressure wound therapy in refractory exit-site and tunnel infections: a single center experience. BMC Nephrol. 2024 Aug 26;25(1):273. doi: 10.1186/s12882-024-03714-8. PMID: 39187762; PMCID: PMC11346171.

***Editor’s comment 2:*** *Add the results of microbial culture****.***

**Response:**

We thank the Editor for this valuable suggestion. However, we regret that microbial culture results were not systematically available in our retrospective multicenter dataset. The primary objective of our study was to evaluate the predictive value of complete blood count (CBC)-based inflammatory markers for the occurrence of peritonitis, which is more central to our research focus than characterizing the microbiological spectrum. In many participating centers, empirical antibiotic therapy was often initiated immediately upon clinical suspicion of peritonitis, and peritoneal effluent cultures were either not consistently obtained or were incompletely documented in the medical records. Therefore, we are unable to provide reliable microbial culture data.

We acknowledge this as a limitation of our study and have added a statement in the **Discussion** section **(page 27, lines 441–445)** to address this issue. Specifically, we now state:
“Microbiological culture results were not systematically available in this retrospective cohort, which limits our ability to assess whether the predictive performance of CBC-derived markers differs by pathogen type. Future prospective studies should incorporate standardized microbial sampling to address this question.”

We hope the Editor and reviewers will accept this clarification. We have transparently highlighted this limitation in the revised manuscript and have refrained from over-interpreting our findings.

***Editor’s comment 3:*** *Recheck the typing of references as guidelines.*

We thank the Editor for this reminder. We have carefully rechecked all references against the journal’s formatting guidelines and corrected any typographical or stylistic errors. The reference list has been revised accordingly in the resubmitted manuscript. We hope that this revision now meets the journal’s requirements.

**Responses to Reviewer #1**

***Reviewer*** ***#1 comment (1. Strengths of the Manuscript):*** *Thank you for providing the manuscript... Below are my detailed comments and suggestions for progression.*

We greatly appreciate the reviewer’s positive assessment of our study’s strengths—namely, the multicenter design, systematic comparison of six markers, and statistical robustness—which has been very helpful in further improving the quality of our manuscript.

***Reviewer #1 comment*** ***(******2. Critical Areas for Improvement A. Methodological Clarifications • Baseline Period Logic******):***

*You defined the baseline as laboratory measurements taken within the first three months of PD initiation and excluded patients who developed peritonitis during this window. Please clarify the clinical rationale for this three-month stable period. While it ensures "stable" baseline values, it may introduce* *immortal time bias or selection bias by excluding early-onset peritonitis cases which are often the most clinically concerning.*

**Response:**

We thank the reviewer for raising this important methodological concern. We fully acknowledge the potential for immortal time bias and selection bias when excluding early-onset peritonitis cases. Below we clarify the clinical rationale for the 3‑month stable period, and we have added a corresponding limitation statement in the Discussion section of the revised manuscript.

**Clinical rationale for the 3‑month landmark period**

The first three months after peritoneal dialysis (PD) initiation are widely recognized as a period of physiological and mechanical instability. During this “break‑in” phase, patients frequently experience catheter‑related complications, exit‑site or tunnel infections, and ongoing peritoneal membrane healing following catheter implantation. These factors can induce transient, non‑steady‑state inflammation that does not reflect the patient’s long‑term inflammatory set point. Consequently, using laboratory values obtained during this early window as a “baseline” may confound the association between chronic inflammatory markers and subsequent peritonitis risk. **Excluding peritonitis episodes that occur during this unstable period and anchoring baseline measurements after the first three months represents a methodologically conservative approach to define a true “steady‑state” baseline risk profile.**

This rationale is directly supported by several studies. Poulain *et al.* explicitly defined early‑onset peritonitis as peritonitis occurring within the first three months on PD, providing a direct precedent for using this threshold to separate early instability from the stable baseline period [1]. Harel *et al.* also defined early‑onset peritonitis as that occurring within three months of catheter insertion and reported significantly higher peritonitis rates during this interval, further confirming the elevated risk in the early period [2]. Similarly, Wu *et al.*, in a large southern Chinese cohort, adopted the ≤3‑month definition and identified specific risk factors for early‑onset peritonitis, independently validating the same landmark threshold [3]. Ma *et al.*, in a multicentre retrospective cohort study, applied strict exclusion criteria based on PD history and categorised peritonitis within six months as early onset, underscoring the methodological importance of defining a landmark period before assessing baseline risk [4]. Furthermore, a systematic review by Zhao *et al.* noted that high bias risks in PD peritonitis prediction models often arise from data source and statistical design issues; our use of a 3‑month landmark before baseline assessment is intended to minimise such bias [5].

**[1]** Poulain L, Bechade C, Lanot A, Ficheux M, Guillouet S, Lobbedez T, Boyer A, Boyer A. Early-Onset Peritonitis and Outcomes of Peritoneal Dialysis: A Cohort Study with Data from the RDPLF. Am J Nephrol. 2025;56(4):391-402. doi: 10.1159/000542835. Epub 2025 Feb 3. PMID: 39900007.

**[2]** Harel Z, Wald R, Bell C, Bargman JM. Outcome of patients who develop early-onset peritonitis. Adv Perit Dial. 2006;22:46-9. PMID: 16983938.

**[3]** Wu H, Huang R, Yi C, Wu J, Guo Q, Zhou Q, Yu X, Yang X. Risk Factors for Early-Onset Peritonitis in Southern Chinese Peritoneal Dialysis Patients. Perit Dial Int. 2016 11-12;36(6):640-646. doi: 10.3747/pdi.2015.00203. Epub 2016 May 4. PMID: 27147289; PMCID: PMC5174871.

**[4]** Ma X, Shi Y, Tao M, Jiang X, Wang Y, Zang X, Fang L, Jiang W, Du L, Jin D, Zhuang S, Liu N. Analysis of risk factors and outcome in peritoneal dialysis patients with early-onset peritonitis: a multicentre, retrospective cohort study. BMJ Open. 2020 Feb 13;10(2):e029949. doi: 10.1136/bmjopen-2019-029949. PMID: 32060152; PMCID: PMC7045164.
**[5]** Zhao Z, Li Y, Quan Q, Wang H, Zhang W, Zhang X. Risk prediction models for peritoneal dialysis-associated peritonitis: a systematic review and meta-analysis. Int Urol Nephrol. 2026 Mar;58(3):967-979. doi: 10.1007/s11255-025-04795-6. Epub 2025 Sep 21. PMID: 40975843.

**Acknowledgment of limitation in the Discussion**

We agree with the reviewer that excluding early-onset peritonitis cases may introduce immortal time bias or selection bias. To address this transparently, we have added a clear limitation statement in the **Discussion** section (**page 27, lines 4****45–454**):

“Our primary analysis excluded patients who developed peritonitis within the first three months after PD initiation. This exclusion was based on a well-established clinical rationale—the early ‘break in’ period is characterized by unique, transient inflammatory drivers that differ from steady state peritonitis risk [33, 34]. Nevertheless, we acknowledge that this approach may introduce immortal time bias and selection bias, and our findings are most applicable to peritonitis risk assessment after the initial three‑month stabilization period. Future prospective studies with serial biomarker collection starting from PD catheter implantation are needed to validate our findings in the very early post PD period”

We sincerely hope this supplementary note properly addresses the methodological limitation, whilst preserving the clinical justification behind our landmark-based study design. We highly appreciate your thoughtful suggestions; your feedback has substantially enhanced the transparency and rigour of our manuscript.

***Reviewer #1 comment (2. Critical Areas for Improvement A. Methodological Clarifications •*** ***Data Collection Dates):***

*There is a potential typo in the Methods section. It states data collection began on 01/07/2025 and ended on 01/10/2025. However, the follow-up period is listed as starting in 1998 and ending in 2023.Ensure the retrospective data extraction dates (2025) are clearly distinguished from the actual clinical follow-up years (1998–2023) to avoid chronological confusion for the reader.*

**Response:**

We thank the reviewer for identifying this potential source of confusion and apologize for any lack of clarity. The dates “July 1, 2025” to “October 1, 2025” refer to the period of retrospective data extraction and database assembly, not the clinical follow-up period. The actual clinical follow-up for patients spanned from July 21, 1998, to May 31, 2023. We have revised the **Methods** section to clearly distinguish these two timeframes.

**Changes made in the manuscript (page 8, lines 144–147):**
“Clinical data were retrospectively collected from the medical records of patients who initiated PD between July 21, 1998, and May 31, 2023. The retrospective data extraction for this study was performed between July 1, 2025, and October 1, 2025”.

***Reviewer #1 comment (2. Critical Areas for Improvement B. Data Synthesis and Interpretation • Comparative Performance):***

*The C-indices for PIV (0.67), SII (0.70), and PLR (0.70) are described as "moderate".
A C-index of 0.70 is acceptable but on the lower end for a predictive "risk stratification" tool. You should discuss whether combining these markers with established clinical risk factors (like albumin or diabetes) significantly improves the model's net reclassification index (NRI).*

**Response:**

We thank the reviewer for the valuable comment regarding the moderate discriminative performance of the indices and the need to discuss net reclassification improvement (NRI).

It should be clarified that the reported C‑indices for PIV (0.67), SII (0.70), and PLR (0.70) were derived from models that already adjusted for established clinical risk factors (including cardiovascular disease, diabetes, sex, age, serum albumin, smoking, hyperlipidemia, high‑sensitivity C‑reactive protein, hemoglobin, and total Kt/V). While these values indicate modest discriminatory ability, they are not indicative of confounding, as all three indices retained independent predictive value in the fully adjusted multivariable Cox regression (Model 3). Nevertheless, we agree that a C‑index of 0.67–0.70 is suboptimal for a stand‑alone risk stratification tool. Therefore, these peripheral blood inflammatory indices are better suited as risk modifiers to complement conventional models rather than as independent predictors.

**Regarding NRI:** We agree with the reviewer that NRI would be an informative metric to assess whether adding PIV, SII, or PLR to conventional risk factors improves risk reclassification. We did not perform NRI in the current study for the following methodological reasons, which we believe are important to state transparently.

First, NRI calculation requires pre‑specified risk categories (e.g., low, intermediate, high) and landmark time points for reclassification. These thresholds were not pre‑defined in our statistical analysis plan, as the primary aim was to evaluate independent prognostic associations rather than to develop a prediction model. Post‑hoc selection of risk strata would introduce arbitrariness and potential bias, even in a large multicenter dataset.

Second, NRI is highly sensitive to the choice of risk thresholds. In large samples, even negligible reclassification improvements can become statistically significant without necessarily translating to clinical utility. Given the modest C‑indices (0.67–0.70) observed, we considered that a post‑hoc NRI analysis might generate misleadingly “positive” results of limited clinical relevance, and we preferred to avoid such overinterpretation.

Third, best practice for NRI requires concurrent reporting of calibration and decision curve analysis (DCA) to assess clinical net benefit, which were beyond the scope of the present study. Performing NRI alone without calibration and DCA would provide an incomplete and potentially biased assessment of model improvement.

Consequently, whether adding PIV, SII, or PLR to conventional risk factors significantly improves net reclassification remains unknown. We have explicitly stated this as a limitation in the revised Discussion and plan to conduct a dedicated model development and validation study (including NRI, calibration, and DCA) in the future, using pre‑specified risk strata and time points.

**Discussion (added to Discussion section of the manuscript,** **page 26, lines 420–427)**

“The C‑indices for PIV (0.67), SII (0.70), and PLR (0.70) showed modest discrimination. Despite this, all three indices retained independent predictive value in the fully adjusted model (Model 3), indicating that their prognostic information is not driven by confounding. Nevertheless, a C‑index of 0.67–0.70 is suboptimal for a stand‑alone risk stratification tool. Hence, these peripheral blood inflammatory indices should be considered risk modifiers that complement conventional models rather than independent risk stratifiers.”

**Limitations (added to the Limitations** **section of the manuscript, pages 28, lines 469–476)**

“We acknowledge that net reclassification improvement (NRI) was not evaluated in the present study. Although Model 3 demonstrated independent predictive value for PIV, SII, and PLR, whether adding these indices to conventional risk factors significantly improves net reclassification remains unknown. This omission is a clear limitation. In subsequent external validation or prospective studies, we plan to perform NRI analyses to determine whether models incorporating PIV, SII, or PLR together with established clinical factors yield clinically meaningful net reclassification gains.”

***Reviewer #1 comment (2. Critical Areas for Improvement B. Data Synthesis and Interpretation •*** ***Exclusion of PMR):***

*The Platelet-to-Monocyte Ratio (PMR) was excluded because it showed no significant difference (p=0.987). Briefly discuss in the "Discussion" section why PMR might have failed where PIV and SII succeeded. Is it possible that the individual contribution of monocytes is only predictive when moderated by neutrophils, as seen in the PIV formula?*

**Response:**

 We thank the reviewer for raising this insightful question. We have added a dedicated discussion on the potential reasons for PMR’s lack of predictive value.

**Proposed mechanisms (****added to Discussion, pages22–24, lines 355–388):**

“In contrast to PIV, SII, and PLR, the platelet-to-monocyte ratio (PMR) showed no significant association with peritonitis risk (p=0.987). Several explanations may account for this finding. First, monocytes play a dual role in peritoneal defense. A growing body of evidence indicates that while monocyte-derived inflammatory macrophages promote peritoneal fibrosis and disease progression, tissue-resident macrophages are rather considered anti-fibrotic [25]. Long-term PD exposure alters resident peritoneal macrophage phenotype, leading to loss of homeostatic and anti-inflammatory functionality while enhancing inflammatory responses to external stimuli, rendering patients sensitive to peritonitis and subsequent fibrosis [25]. Thus, the net effect of monocytes may depend on their interaction with other immune cells rather than on their absolute count alone. PIV explicitly incorporates neutrophil count (neutrophil × platelet × monocyte / lymphocyte), whereas PMR treats monocytes as an isolated denominator and therefore fails to capture this complex modulation. Second, neutrophils and monocytes exhibit intricate functional interactions in peritoneal inflammation. During septic peritonitis, neutrophils suppress peritoneal inflammatory monocytes through IL-10-mediated regulation, with in vitro experiments confirming that monocyte suppression is mediated by neutrophil-derived IL-10 [26]. This suggests that the inflammatory potential of monocytes is actively modulated by the concomitant neutrophil response—a biological nuance that PIV captures through its multiplicative structure, but PMR, as a simple ratio, does not. Third, PMR’s lack of predictive value may also reflect methodological limitations of the ratio itself. A low PMR could reflect either low platelet count or high monocyte count; the latter might indicate a protective inflammatory response rather than a pathogenic one, leading to opposing clinical interpretations from the same numerical value. Moreover, the optimal balance between platelets and monocytes may vary across individuals, and a simple ratio may not capture the complex interplay among multiple cell lineages. Consistent with this notion, a recent study in PD patients demonstrated that SII—a composite inflammatory index structurally analogous to PIV—was an independent risk factor for frequent PD associated peritonitis, and combining SII with C reactive protein (CRP)/high density lipoprotein cholesterol (HDL C) yielded better predictive performance than single markers [27]. This finding supports our conclusion that the predictive signal carried by monocytes is only apparent when modulated by neutrophil and lymphocyte counts, as operationalized in the PIV formula.”

***Reviewer #1 comment (2. Critical Areas for Improvement*** ***C. Clinical Significance and Application • Practical Thresholds):***

*You identified "optimal cut-offs" via ROC analysis.For these markers to be useful to clinicians, the manuscript needs to emphasize the specificity and sensitivity at these cut-off points. If the sensitivity is low, the markers might miss many high-risk patients; if specificity is low, they might cause unnecessary alarm.*

**Response:**

We sincerely thank the reviewer for this critical and clinically insightful comment. We completely agree that reporting cut-off values without explicit emphasis on sensitivity and specificity limits clinical applicability, and that low sensitivity or low specificity may lead to missed high-risk patients or unnecessary alarm, respectively.

In our original manuscript, we had already reported sensitivity and specificity for each marker in **Table 2** and, importantly, applied these performance characteristics to guide marker selection. Specifically, as described in the **Results** section:

“ROC analysis revealed that all inflammatory markers showed limited discriminatory power for peritonitis (area under the curve [AUC], 0.55–0.59). PIV demonstrated the highest predictive value (AUC = 0.59, p< 0.001). The markers displayed distinct sensitivity–specificity profiles: MLR had high sensitivity (0.73) but low specificity (0.39), whereas PLR showed high specificity (0.86) but low sensitivity (0.27). SII presented a more balanced profile (sensitivity 0.46, specificity 0.72). NLR did not reach statistical significance (AUC = 0.55, p = 0.062). Due to its very low specificity, MLR was considered clinically impractical owing to a high false-positive rate. Consequently, both NLR and MLR were excluded from further analyses (Table 2).

”

In the **Discussion**, we have added a dedicated paragraph addressing the reviewer’s concerns about missed cases and unnecessary alarms（**pages 24–25, lines 38****9–401）**:

“The clinical utility of a predictive marker depends critically on its sensitivity–specificity balance. In our cohort, PLR’s low sensitivity (27%) implies that using this cut-off alone would fail to identify most patients who subsequently develop peritonitis, potentially leading to under-recognition of high-risk individuals. Conversely, its high specificity (86%) means that a positive result is unlikely to be a false alarm. For SII and PIV, the moderate sensitivity and specificity suggest they may serve as adjunctive rather than standalone tests. Importantly, we explicitly excluded MLR from further analyses because its very low specificity (39%) would result in an unacceptably high false-positive rate, causing unnecessary patient anxiety and potentially unwarranted clinical interventions. Therefore, in current clinical practice, we recommend that these markers be interpreted in combination with other clinical parameters, and that dynamic changes over time be considered to improve predictive accuracy.”

***Reviewer #1 comment (2. Critical Areas for Improvement C. Clinical Significance and Application • Long-term vs. Short-term Prediction):***

*Since markers were taken at PD initiation, how stable are these values over years of dialysis? Address whether a single baseline measurement is sufficient for a median follow-up of 49 months, or if a "time-dependent" Cox model would have better captured the risk as inflammatory status changes over time.*

**Response:**

We thank the reviewer for this insightful and clinically relevant comment. We fully agree that inflammatory markers may change over time on peritoneal dialysis (PD), and that a time-dependent Cox model would theoretically provide a more dynamic assessment of risk. We address the reviewer’s concern in three parts below, and have added corresponding content to the Discussion section of the revised manuscript.

Firstly，we recognize that a single baseline measurement—taken at PD initiation or within the first three months—cannot fully capture the longitudinal evolution of inflammatory status over a median follow-up of 49 months. Due to the retrospective nature of our multicenter cohort study, we did not systematically collect serial complete blood count data at prespecified intervals after baseline. Therefore, we are unable to perform a time-dependent Cox regression analysis that would require repeated measurements of the inflammatory markers over time. This is an important limitation, which we have now explicitly stated in the revised Discussion section.

Secondly, despite this limitation, we would like to clarify that our study was specifically designed to evaluate the predictive value of baseline inflammatory markers—that is, the ability of markers measured at or near PD initiation to stratify subsequent peritonitis risk. This research question is clinically meaningful and methodologically well-established in the field of prognostic marker research. A single baseline measurement reflects the patient’s immuno-inflammatory set‑point at the time of dialysis transition, which may have independent prognostic significance irrespective of later fluctuations. Some landmark cohort studies have successfully used baseline measurements to predict long-term outcomes (e.g., mortality) in PD patients [1,2]. and our approach is consistent with those works. Thus, while acknowledging its limitations, we maintain that our study provides valid and clinically useful information regarding the role of initial inflammatory status in predicting future peritonitis risk.

Thirdly, we fully agree with the reviewer that a time-dependent Cox model would better capture the changing risk as inflammatory status evolves over years of dialysis. We have therefore added a clear recommendation in the Discussion section that future prospective studies should include serial measurements of CBC-based inflammatory markers (e.g., every 3–6 months) to allow time-dependent analyses. Such studies could also investigate whether changes in these markers (e.g., a rising PIV or SII) provide incremental predictive value beyond baseline values.

Changes made in the revised manuscript **(the Limitations subsection of the Discussion section, pages 27–28, lines 454–469):**

We have added the following new paragraph:

“We did not collect serial complete blood count data during follow-up and therefore could not perform time-dependent Cox regression analyses. A single baseline measurement, while reflecting the patient’s initial inflammatory set‑point at PD transition, may not capture subsequent changes in inflammatory status over a median follow‑up of 49 months. We acknowledge that inflammatory markers can fluctuate over time in response to intercurrent infections, changes in dialysis prescription, or nutritional status. Nevertheless, evaluating the predictive value of baseline markers is a clinically meaningful and methodologically accepted approach in prognostic research, and our findings demonstrate that even a one‑time measurement provides useful risk stratification. The lack of repeated measurements precludes assessment of whether dynamic changes in these markers (e.g., a rising PIV or SII) add further predictive value. Future prospective studies with standardized serial sampling (e.g., every 3–6 months) are needed to validate the utility of time‑dependent inflammatory marker monitoring and to determine whether incorporating longitudinal data improves peritonitis risk prediction over baseline assessment alone”

[1] Damarlapally N, Vempati R, Mourad D, Vasudevan SS, Mathur G, Khan A, Banda P, Polamarasetty H, Chauhan S, Manjappachar N, Bhatti T, Desai R. Association of neutrophil-lymphocyte ratio with all-cause and cardiovascular mortality in peritoneal dialysis patients: A systematic review and meta-analysis. World J Methodol. 2026 Mar 20;16(1):106277. doi: 10.5662/wjm.v16.i1.106277. PMID: 41809153; PMCID: PMC12968771.

[2]Li G, Yu J, Jiang S, Wu K, Xu Y, Lu X, Wang Y, Lin J, Yang X, Li Z, Mao H. Systemic Immune-Inflammation Index Was Significantly Associated with All-Cause and Cardiovascular-Specific Mortalities in Patients Receiving Peritoneal Dialysis. J Inflamm Res. 2023 Aug 31;16:3871-3878. doi: 10.2147/JIR.S426961. PMID: 37671129; PMCID: PMC10476662.

***Reviewer #1 comment (3. Technical and Formal Suggestions*** ***• Reporting Guidelines):***

*Ensure the manuscript explicitly states adherence to the STROBE (Strengthening the Reporting of Observational Studies in Epidemiology) guidelines, as is standard for cohort studies in journals like PLOS ONE.*

**Response:**

We thank the reviewer for this valuable suggestion. We have added a clear statement on **page 8, lines 152-154** in the **Methods** section: “This study adheres to the Strengthening the Reporting of Observational Studies in Epidemiology (STROBE) guidelines for cohort studies.” The completed STROBE checklist has been uploaded as an “Other” file in the submission system.

***Reviewer #1 comment (3. Technical and Formal Suggestions*** ***• Language and Flow):***

*The abstract is well-structured, but the transition between the individual ratio descriptions in the introduction could be tightened to focus more on why these specific composite markers (like PIV) are theoretically superior to simple ratios.*

**Response:**

We sincerely thank the reviewer for this constructive and insightful comment. We agree that a clearer theoretical justification for the use of composite markers (e.g., PIV) over simple ratios would improve the logical flow and scientific rigor of the introduction. Accordingly, we have revised the **Introduction** section to enhance the transition between descriptions of individual inflammatory ratios and to explicitly highlight the theoretical advantages of composite markers. The modifications have been made on **pages 5-6, lines 87–111** of the revised manuscript.

“In this context, inflammatory markers derived from peripheral blood cell counts have attracted growing interest for their prognostic utility. Among these, composite indices that integrate multiple cell lineages—such as the pan-immune-inflammatory value (PIV), which combines neutrophil, platelet, monocyte, and lymphocyte counts, and the systemic immune-inflammation index (SII), based on neutrophil, lymphocyte, and platelet counts—offer a more comprehensive reflection of systemic immune-inflammatory status than single-ratio markers. Theoretically, these multicellular indices capture complex interactions between innate and adaptive immunity, as well as thrombotic pathways, thereby providing a broader and potentially more accurate risk assessment (5-7). In contrast, simpler ratios, including the platelet-to-lymphocyte ratio (PLR), monocyte-to-lymphocyte ratio (MLR), neutrophil-to-lymphocyte ratio (NLR), and platelet-to-monocyte ratio (PMR), while readily available and clinically useful in various settings (8-13), may lack the integrated information afforded by composite indices. Notably, PLR and SII have already been applied in the setting of PDAP (9), and MLR and NLR have shown associations with mortality and cardiovascular events in PD patients (10, 11). Theoretically, PIV offers superior discriminative capacity because it simultaneously reflects the pro-inflammatory burden (neutrophils, platelets, monocytes) and the adaptive immune reserve (lymphocytes), thereby providing a more comprehensive assessment of systemic inflammation and immune dysregulation.

Therefore, the extent to which these CBC-derived inflammatory indices can predict PDAP remains largely unknown. To address this gap, we conducted a multicenter retrospective study to systematically assess and compare the predictive performance of several such indices, with the goal of identifying the most effective biomarker for clinical risk stratification in PDAP.”

***Reviewer #1 comment (3. Technical and Formal Suggestions):***

*Re write, editing and grammatical correction and some editing because some paragraph simple copy past from AI.*

**Response:**

Thank you for your careful and attentive review of our manuscript. We sincerely apologize for the language issues and the inappropriate use of AI‑generated text in our original submission, and we would like to take this opportunity to explain the circumstances. Prior to submission, we had engaged a professional editing company to polish the manuscript. However, due to a communication error with the editing service, we did not receive the final proofread version before the submission. Consequently, we inadvertently uploaded the unedited raw manuscript.

We have since obtained the professionally edited version and have carefully revised the entire manuscript accordingly. The following materials are being uploaded with this revision:

1. **Revised manuscript (all changes highlighted)** – Major revisions include grammar corrections, sentence restructuring, rewriting of problematic paragraphs, and removal of all unmodified AI‑generated text. Given the extensive scope of these changes, we have highlighted all revisions directly in the manuscript rather than listing them individually.
2. **Professional editing certificate (attached as “Other” file)** – The editing service was provided by Expert Editing ([exediting.com](https://exediting.com/)). The details are as follows:
   - Website: [http://www.exediting.com](http://www.exediting.com/)
   - Email: service@exediting.com
   - Certificate No.: EE-260104-FA-0000027-W5O

We hope that the language quality of the revised manuscript now meets the journal’s standards. Should you, upon your review, find any remaining language issues or have further suggestions for improvement, please do not hesitate to let us know, and we will promptly carry out additional revisions.

Thank you again for your patience and professional guidance.

**Responses to Reviewer #2**

***Reviewer #2 comment:*** *Interesting piece of work. Well presented. Appropriate statistical anamysis with good graph to prove the subject of study.* *S**ome minor typo errors need addressining. All relevant data provided and easy to understant in the manuscript. Approptiate references taken into consideration.*

**Response:**

We thank the reviewer very much for the positive assessment of our work. We are pleased that the reviewer found the study interesting, well presented, with appropriate statistical analysis, clear graphs, accessible data, and relevant references. We have also carefully addressed the reviewer’s note regarding minor typographical errors. The entire manuscript has been rechecked line by line, and all typographical mistakes have been corrected. The revisions are marked in the updated manuscript.

We appreciate the reviewer’s time and insightful suggestions, which have helped us improve the clarity and accuracy of our paper.
