# Supplementary material for: Predictive value of complete blood cell count-based inflammatory markers for peritonitis risk in peritoneal dialysis patients: A multicenter cohort study
Source: PLoS One. 2026 Jul 22;21(7):e0354120. doi: 10.1371/journal.pone.0354120 (PMC13390817; doi:10.1371/journal.pone.0354120)
Supplement: Peer review history

# Author comment: Author response to Decision Letter 0

**DOI:** 10.1371/journal.pone.0354120.r001

12 Feb 2026

Attachment Submitted filename: Response to Reviewers.docx

# Peer review: Decision Letter 0

**DOI:** 10.1371/journal.pone.0354120.r002

**Authors:** Marwan Al-Nimer

15 May 2026

PONE-D-26-05780

Predictive Values of Complete Blood Cell Count-Based Inflammatory Markers for Peritonitis Risk in Peritoneal Dialysis Patients: A Multicenter Cohort Study

PLOS One

Dear Dr. Tian,

Thank you for submitting your manuscript to PLOS ONE. After careful consideration, we feel that it has merit but does not fully meet PLOS ONE’s publication criteria as it currently stands. Therefore, we invite you to submit a revised version of the manuscript that addresses the points raised during the review process.

==============================

**ACADEMIC EDITOR: Minor revision**

==============================

Please submit your revised manuscript by Jun 29 2026 11:59PM. If you will need more time than this to complete your revisions, please reply to this message or contact the journal office at plosone@plos.org. When you're ready to submit your revision, log on to https://www.editorialmanager.com/pone/ and select the 'Submissions Needing Revision' folder to locate your manuscript file.

Please include the following items when submitting your revised manuscript:

- A letter that responds to each point raised by the academic editor and reviewer(s). You should upload this letter as a separate file labeled 'Response to Reviewers'.
- A marked-up copy of your manuscript that highlights changes made to the original version. You should upload this as a separate file labeled 'Revised Manuscript with Track Changes'.
- An unmarked version of your revised paper without tracked changes. You should upload this as a separate file labeled 'Manuscript'.

If you would like to make changes to your financial disclosure, please include your updated statement in your cover letter. Guidelines for resubmitting your figure files are available below the reviewer comments at the end of this letter.

If applicable, we recommend that you deposit your laboratory protocols in protocols.io to enhance the reproducibility of your results. Protocols.io assigns your protocol its own identifier (DOI) so that it can be cited independently in the future. For instructions see: https://journals.plos.org/plosone/s/submission-guidelines#loc-laboratory-protocols. Additionally, PLOS ONE offers an option for publishing peer-reviewed Lab Protocol articles, which describe protocols hosted on protocols.io. Read more information on sharing protocols at https://plos.org/protocols?utm_medium=editorial-email&utm_source=authorletters&utm_campaign=protocols.

As the corresponding author, your ORCID iD is verified in the submission system and will appear in the published article. PLOS supports the use of ORCID, and we encourage all coauthors to register for an ORCID iD and use it as well. Please encourage your coauthors to verify their ORCID iD within the submission system before final acceptance, as unverified ORCID iDs will not appear in the published article. *Only*  the individual author can complete the verification step; PLOS staff *cannot*  verify ORCID iDs on behalf of authors.

We look forward to receiving your revised manuscript.

Kind regards,

Marwan Salih Al-Nimer, MD, PhD

Academic Editor

PLOS One

Journal Requirements:

When submitting your revision, we need you to address these additional requirements.

1. Please ensure that your manuscript meets PLOS ONE's style requirements, including those for file naming. The PLOS ONE style templates can be found at https://journals.plos.org/plosone/s/file?id=wjVg/PLOSOne_formatting_sample_main_body.pdf and https://journals.plos.org/plosone/s/file?id=ba62/PLOSOne_formatting_sample_title_authors_affiliations.pdf

2. Please note that PLOS One has specific guidelines on code sharing for submissions in which author-generated code underpins the findings in the manuscript. In these cases, all author-generated code must be made available without restrictions upon publication of the work. Please review our guidelines at https://journals.plos.org/plosone/s/materials-and-software-sharing#loc-sharing-code and ensure that your code is shared in a way that follows best practice and facilitates reproducibility and reuse.

3. Thank you for stating in your Funding Statement:

[The author(s) declare that financial support was received for the research and/or publication of this article. This study was supported by the Ningxia Natural Science Foundation (Key Project, 2022AAC02062), the Ningxia Natural Science Foundation (2023AACO3568), the Key R&D Program of Ningxia Autonomous Region (2022BEG03120), and the National Natural Science Foundation of China (No. 82360153).].

Please provide an amended statement that declares *all* the funding or sources of support (whether external or internal to your organization) received during this study, as detailed online in our guide for authors at http://journals.plos.org/plosone/s/submit-now.  Please also include the statement “There was no additional external funding received for this study.” in your updated Funding Statement.

Please include your amended Funding Statement within your cover letter. We will change the online submission form on your behalf.

4. Please remove any funding-related text from the manuscript. Please note that funding information should not appear in any section or other areas of your manuscript. We will publish the funding statement that is present in the Financial Disclosure section of the online submission form.

5. In the online submission form you indicate that your data is not available for proprietary reasons and have provided a contact point for accessing this data. Please note that your current contact point is a co-author on this manuscript. According to our Data Policy, the contact point must not be an author on the manuscript and must be an institutional contact, ideally not an individual. Please revise your data statement to a non-author institutional point of contact, such as a data access or ethics committee, and send this to us via return email. Please also include contact information for the third party organization, and please include the full citation of where the data can be found.

6. Please include your full ethics statement in the ‘Methods’ section of your manuscript file. In your statement, please include the full name of the IRB or ethics committee who approved or waived your study, as well as whether or not you obtained informed written or verbal consent. If consent was waived for your study, please include this information in your statement as well.

7. Please upload a new copy of Figures 1-5 as the detail is not clear. Please follow the link for more information:  https://journals.plos.org/plosone/s/figures

If the reviewer comments include a recommendation to cite specific previously published works, please review and evaluate these publications to determine whether they are relevant and should be cited. There is no requirement to cite these works unless the editor has indicated otherwise.

Please review your reference list to ensure that it is complete and correct. If you have cited papers that have been retracted, please include the rationale for doing so in the manuscript text, or remove these references and replace them with relevant current references. Any changes to the reference list should be mentioned in the rebuttal letter that accompanies your revised manuscript. If you need to cite a retracted article, indicate the article’s retracted status in the References list and also include a citation and full reference for the retraction notice.

Additional Editor Comments:

Recheck the sentence "white blood cell count >100/μL" page 8 Line 133.

Add the results of microbial culture

Recheck the typing of references as guidelines

[Note: HTML markup is below. Please do not edit.]

Reviewers' comments:

Reviewer's Responses to Questions

**Comments to the Author**

1. Is the manuscript technically sound, and do the data support the conclusions?

The manuscript must describe a technically sound piece of scientific research with data that supports the conclusions. Experiments must have been conducted rigorously, with appropriate controls, replication, and sample sizes. The conclusions must be drawn appropriately based on the data presented.

Reviewer #1: Yes

Reviewer #2: Yes

**********

2. Has the statistical analysis been performed appropriately and rigorously?

Reviewer #1: Yes

Reviewer #2: Yes

**********

3. Have the authors made all data underlying the findings in their manuscript fully available?

The PLOS Data policy requires authors to make all data underlying the findings described in their manuscript fully available without restriction, with rare exception (please refer to the Data Availability Statement in the manuscript PDF file). The data should be provided as part of the manuscript or its supporting information, or deposited to a public repository. For example, in addition to summary statistics, the data points behind means, medians and variance measures should be available. If there are restrictions on publicly sharing data—e.g. participant privacy or use of data from a third party—those must be specified.

Reviewer #1: No

Reviewer #2: Yes

**********

4. Is the manuscript presented in an intelligible fashion and written in standard English?

PLOS ONE does not copyedit accepted manuscripts, so the language in submitted articles must be clear, correct, and unambiguous. Any typographical or grammatical errors should be corrected at revision, so please note any specific errors here.

Reviewer #1: Yes

Reviewer #2: Yes

**********

5. Review Comments to the Author

Please use the space provided to explain your answers to the questions above. You may also include additional comments for the author, including concerns about dual publication, research ethics, or publication ethics. (Please upload your review as an attachment if it exceeds 20,000 characters)

Reviewer #1: Thank you for providing the manuscript. I have conducted a review of your study, "Predictive Values of Complete Blood Cell Count-Based Inflammatory Markers for Peritonitis Risk in Peritoneal Dialysis Patients: A Multicenter Cohort Study". ID: PONE-D-26-05780

As a reviewer, I have evaluated the manuscript based on methodological rigor, data synthesis, and clinical significance. Below are my detailed comments and suggestions for progression.

1. Strengths of the Manuscript

• Scale and Scope: The use of a multicenter cohort involving 2,036 patients across 10 centers provides significant statistical power and enhances the external validity of the findings.

• Novelty in Comparison: The study systematically compares six different CBC-derived markers (PIV, SII, PLR, NLR, MLR, and PMR), which adds value beyond existing studies that often focus on only one or two indices.

• Statistical Robustness: The use of restricted cubic splines to identify nonlinear associations and multivariate Cox regression for independent risk assessment is appropriate for this type of clinical data.

2. Critical Areas for Improvement

A. Methodological Clarifications

• Baseline Period Logic: You defined the baseline as laboratory measurements taken within the first three months of PD initiation and excluded patients who developed peritonitis during this window.

o Comment: Please clarify the clinical rationale for this three-month stable period. While it ensures "stable" baseline values, it may introduce immortal time bias or selection bias by excluding early-onset peritonitis cases which are often the most clinically concerning.

• Data Collection Dates: There is a potential typo in the Methods section. It states data collection began on 01/07/2025 and ended on 01/10/2025. However, the follow-up period is listed as starting in 1998 and ending in 2023.

o Recommendation: Ensure the retrospective data extraction dates (2025) are clearly distinguished from the actual clinical follow-up years (1998–2023) to avoid chronological confusion for the reader.

B. Data Synthesis and Interpretation

• Comparative Performance: The C-indices for PIV (0.67), SII (0.70), and PLR (0.70) are described as "moderate".

o Comment: A C-index of 0.70 is acceptable but on the lower end for a predictive "risk stratification" tool. You should discuss whether combining these markers with established clinical risk factors (like albumin or diabetes) significantly improves the model's net reclassification index (NRI).

• Exclusion of PMR: The Platelet-to-Monocyte Ratio (PMR) was excluded because it showed no significant difference (p=0.987).

o Comment: Briefly discuss in the "Discussion" section why PMR might have failed where PIV and SII succeeded. Is it possible that the individual contribution of monocytes is only predictive when moderated by neutrophils, as seen in the PIV formula?

C. Clinical Significance and Application

• Practical Thresholds: You identified "optimal cut-offs" via ROC analysis.

o Comment: For these markers to be useful to clinicians, the manuscript needs to emphasize the specificity and sensitivity at these cut-off points. If the sensitivity is low, the markers might miss many high-risk patients; if specificity is low, they might cause unnecessary alarm.

o Long-term vs. Short-term Prediction: Since markers were taken at PD initiation, how stable are these values over years of dialysis? Address whether a single baseline measurement is sufficient for a median follow-up of 49 months, or if a "time-dependent" Cox model would have better captured the risk as inflammatory status changes over time.

3. Technical and Formal Suggestions

• Reporting Guidelines: Ensure the manuscript explicitly states adherence to the STROBE (Strengthening the Reporting of Observational Studies in Epidemiology) guidelines, as is standard for cohort studies in journals like PLOS ONE.

• Language and Flow: The abstract is well-structured, but the transition between the individual ratio descriptions in the introduction could be tightened to focus more on why these specific composite markers (like PIV) are theoretically superior to simple ratios.

• Re write, editing and grammatical correction and some editing because some paragraph simple copy past from AI.

Overall Verdict: This is a strong, well-powered study. Addressing the "immortal time" concern and clarifying the data collection timeline will significantly strengthen the manuscript for final acceptance.

Reviewer #2: Interesting piece of work

Well presented.

Appropriate statistical anamysis with good graph to prove the subject of study.

Some minor typo errors need addressining.

All relevant data provided and easy to understant in the manuscript.

Approptiate references taken into consideration.

**********

6. PLOS authors have the option to publish the peer review history of their article (what does this mean?). If published, this will include your full peer review and any attached files.

If you choose “no”, your identity will remain anonymous but your review may still be made public.

**Do you want your identity to be public for this peer review?**  For information about this choice, including consent withdrawal, please see our Privacy Policy.

Reviewer #1: No

Reviewer #2: **Yes:** Habib Akbani

**********

[NOTE: If reviewer comments were submitted as an attachment file, they will be attached to this email and accessible via the submission site. Please log into your account, locate the manuscript record, and check for the action link "View Attachments". If this link does not appear, there are no attachment files.]

To ensure your figures meet our technical requirements, please review our figure guidelines: https://journals.plos.org/plosone/s/figures

You may also use PLOS’s free figure tool, NAAS, to help you prepare publication quality figures: https://journals.plos.org/plosone/s/figures#loc-tools-for-figure-preparation.

NAAS will assess whether your figures meet our technical requirements by comparing each figure against our figure specifications.

# Author comment: Author response to Decision Letter 1

**DOI:** 10.1371/journal.pone.0354120.r003

30 May 2026

All comments from the editor and reviewers have been carefully addressed. A detailed point-by-point response letter is provided as a separate uploaded file.

Attachment Submitted filename: Response_to_Reviewers_auresp_1.docx

# Peer review: Decision Letter 1

**DOI:** 10.1371/journal.pone.0354120.r004

**Authors:** Marwan Al-Nimer

5 Jul 2026

Predictive Values of Complete Blood Cell Count-Based Inflammatory Markers for Peritonitis Risk in Peritoneal Dialysis Patients: A Multicenter Cohort Study

PONE-D-26-05780R1

Dear Dr. Na Tian,

We’re pleased to inform you that your manuscript has been judged scientifically suitable for publication and will be formally accepted for publication once it meets all outstanding technical requirements.

Within one week, you’ll receive an e-mail detailing the required amendments. When these have been addressed, you’ll receive a formal acceptance letter and your manuscript will be scheduled for publication.

An invoice will be generated when your article is formally accepted. Please note, if your institution has a publishing partnership with PLOS and your article meets the relevant criteria, all or part of your publication costs will be covered. Please make sure your user information is up-to-date by logging into Editorial Manager at Editorial Manager® and clicking the ‘Update My Information' link at the top of the page. For questions related to billing, please contact billing support.

If your institution or institutions have a press office, please notify them about your upcoming paper to help maximize its impact. If they’ll be preparing press materials, please inform our press team as soon as possible -- no later than 48 hours after receiving the formal acceptance. Your manuscript will remain under strict press embargo until 2 pm Eastern Time on the date of publication. For more information, please contact onepress@plos.org.

Kind regards,

Marwan Salih Al-Nimer, MD, PhD

Academic Editor

PLOS One

Additional Editor Comments (optional):

Reviewers' comments:

Reviewer's Responses to Questions

**Comments to the Author**

1. If the authors have adequately addressed your comments raised in a previous round of review and you feel that this manuscript is now acceptable for publication, you may indicate that here to bypass the “Comments to the Author” section, enter your conflict of interest statement in the “Confidential to Editor” section, and submit your "Accept" recommendation.

Reviewer #3: (No Response)

**********

2. Is the manuscript technically sound, and do the data support the conclusions?

The manuscript must describe a technically sound piece of scientific research with data that supports the conclusions. Experiments must have been conducted rigorously, with appropriate controls, replication, and sample sizes. The conclusions must be drawn appropriately based on the data presented.

Reviewer #3: Partly

**********

3. Has the statistical analysis been performed appropriately and rigorously?

Reviewer #3: Yes

**********

4. Have the authors made all data underlying the findings in their manuscript fully available?

The PLOS Data policy requires authors to make all data underlying the findings described in their manuscript fully available without restriction, with rare exception (please refer to the Data Availability Statement in the manuscript PDF file). The data should be provided as part of the manuscript or its supporting information, or deposited to a public repository. For example, in addition to summary statistics, the data points behind means, medians and variance measures should be available. If there are restrictions on publicly sharing data—e.g. participant privacy or use of data from a third party—those must be specified.

Reviewer #3: Yes

**********

5. Is the manuscript presented in an intelligible fashion and written in standard English?

PLOS ONE does not copyedit accepted manuscripts, so the language in submitted articles must be clear, correct, and unambiguous. Any typographical or grammatical errors should be corrected at revision, so please note any specific errors here.

Reviewer #3: Yes

**********

6. Review Comments to the Author

Please use the space provided to explain your answers to the questions above. You may also include additional comments for the author, including concerns about dual publication, research ethics, or publication ethics. (Please upload your review as an attachment if it exceeds 20,000 characters)

Reviewer #3: 1. Page 3, line 54: The term “survival differences” should be revised to “peritonitis-free survival,” which would be more precise in the context of this study.

2. Page 9, lines 160–161 state that “Patients who developed peritonitis during this three-month baseline period were excluded.” Please clarify whether these patients were excluded before or after laboratory data collection. If patients were enrolled and had baseline laboratory data collected first, but were subsequently excluded because they developed PD-associated peritonitis within the following three months, this approach may lead to selection bias. Although the rationale for this exclusion is discussed and supported by previous studies, excluding patients who develop early PDAP after enrollment may remove the events most likely to be predicted by baseline inflammatory indices, namely near-term infection-related inflammatory risk. Therefore, I would not recommend excluding patients who developed PDAP within three months after enrollment or PD initiation from the primary analysis.

3. Table 1 does not present p values, or the p values may be outside the visible page margin. Please resubmit or reformat Table 1 to ensure that all p values are clearly displayed.

4. The follow-up duration in this study was very long, with a median follow-up of 49.47 months. Because CBC-derived inflammatory indices are dynamic biomarkers, using only baseline inflammatory indices to predict PDAP may be affected by attenuation of their time-related predictive relevance. The authors are encouraged to provide the timing of the first episode of PD-associated peritonitis. If most peritonitis episodes occurred late during follow-up, the biological plausibility of the association between baseline inflammatory indices and subsequent peritonitis would be weaker.

**********

7. PLOS authors have the option to publish the peer review history of their article (what does this mean?). If published, this will include your full peer review and any attached files.

If you choose “no”, your identity will remain anonymous but your review may still be made public.

**Do you want your identity to be public for this peer review?**  For information about this choice, including consent withdrawal, please see our Privacy Policy.

Reviewer #3: No

**********

# Editor report: Acceptance letter

**DOI:** 10.1371/journal.pone.0354120.r005

**Authors:** Marwan Al-Nimer

PONE-D-26-05780R1

PLOS One

Dear Dr. Tian,

I'm pleased to inform you that your manuscript has been deemed suitable for publication in PLOS One. Congratulations! Your manuscript is now being handed over to our production team.

At this stage, our production department will prepare your paper for publication. This includes ensuring the following:

* All references, tables, and figures are properly cited

* All relevant supporting information is included in the manuscript submission,

* There are no issues that prevent the paper from being properly typeset

You will receive further instructions from the production team, including instructions on how to review your proof when it is ready. Please keep in mind that we are working through a large volume of accepted articles, so please give us a few days to review your paper and let you know the next and final steps.

Lastly, if your institution or institutions have a press office, please let them know about your upcoming paper now to help maximize its impact. If they'll be preparing press materials, please inform our press team within the next 48 hours. Your manuscript will remain under strict press embargo until 2 pm Eastern Time on the date of publication. For more information, please contact onepress@plos.org.

You will receive an invoice from PLOS for your publication fee after your manuscript has reached the completed accept phase. If you receive an email requesting payment before acceptance or for any other service, this may be a phishing scheme. Learn how to identify phishing emails and protect your accounts at https://explore.plos.org/phishing.

If we can help with anything else, please email us at customercare@plos.org.

Thank you for submitting your work to PLOS ONE and supporting open access.

Kind regards,

PLOS ONE Editorial Office Staff

on behalf of

Professor Marwan Salih Al-Nimer

Academic Editor

PLOS One
